# BZU2/ZmMUTE controls symmetrical division of guard mother cell and specifies neighbor cell fate in maize

**DOI:** 10.1371/journal.pgen.1008377

**Published:** 2019-08-29

**Authors:** Hongliang Wang, Siyi Guo, Xin Qiao, Jianfei Guo, Zuliang Li, Yusen Zhou, Shenglong Bai, Zhiyong Gao, Daojie Wang, Pengcheng Wang, David W. Galbraith, Chun-Peng Song

**Affiliations:** 1 Key Laboratory of Plant Stress Biology, School of Life Sciences, Henan University, Kaifeng, China; 2 State Key Laboratory of Cotton Biology, School of Life Sciences, Henan University, Kaifeng, China; 3 Shanghai Center for Plant Stress Biology, CAS Center for Excellence in Molecular Plant Sciences, Chinese Academy of Sciences, Shanghai, China; 4 School of Plant Sciences, the University of Arizona, Tucson, Arizona, United States of America; "USDA-ARS Pacific West Area", UNITED STATES

## Abstract

Intercellular communication in adjacent cell layers determines cell fate and polarity, thus orchestrating tissue specification and differentiation. Here we use the maize stomatal apparatus as a model to investigate cell fate determination. Mutations in *ZmBZU2* (*b**i**zu**i2*, *bzu2*) confer a complete absence of subsidiary cells (SCs) and normal guard cells (GCs), leading to failure of formation of mature stomatal complexes. Nuclear polarization and actin accumulation at the interface between subsidiary mother cells (SMCs) and guard mother cells (GMCs), an essential pre-requisite for asymmetric cell division, did not occur in *Zmbzu2* mutants. *ZmBZU2* encodes a basic helix-loop-helix (bHLH) transcription factor, which is an ortholog of AtMUTE in *Arabidopsis* (BZU2/ZmMUTE). We found that a number of genes implicated in stomatal development are transcriptionally regulated by BZU2/ZmMUTE. In particular, BZU2/ZmMUTE directly binds to the promoters of *PAN1* and *PAN2*, two early regulators of protodermal cell fate and SMC polarization, consistent with the low levels of transcription of these genes observed in *bzu2-1* mutants. BZU2/ZmMUTE has the cell-to-cell mobility characteristic similar to that of BdMUTE in *Brachypodium distachyon*. Unexpectedly, BZU2/ZmMUTE is expressed in GMC from the asymmetric division stage to the GMC division stage, and especially in the SMC establishment stage. Taken together, these data imply that BZU2/ZmMUTE is required for early events in SMC polarization and differentiation as well as for the last symmetrical division of GMCs to produce the two GCs, and is a master determinant of the cell fate of its neighbors through cell-to-cell communication.

## Author summary

In the grasses, individual stomatal complexes comprise a pair of dumbbell-shaped guard cells associated with two subsidiary cells and the pore, which together play essential roles in the exchange of CO_2_ and O_2_, in xylem transport, and in transpiration. However, little is known about grass stomatal complex development. We have uncovered and characterized a key factor (BZU2/ZmMUTE) determining the formation of guard cells in *Zea mays*. Our data suggest that BZU2/ZmMUTE has a dual role, both as an important player in determining the formation of the guard mother cell, as well as being required for polarization and recruitment of the subsidiary mother cells.

## Introduction

The development of the stomatal complex in maize (*Zea mays*), consisting of a pair of dumbbell-shaped guard cells (GCs) flanked by two subsidiary cells (SCs), provides an excellent model system to study the signals controlling fate determination of adjacent cells by intercellular communication. Unlike the two kidney-shaped GCs in eudicots, the four-celled stomatal complex in grasses may facilitate a faster response to environmental cues in order to optimize photosynthesis and water use [1, 2]. Highly differentiated GCs and SCs in grasses are generated by two asymmetric divisions [3]. The first asymmetric division generates a guard mother cell (GMC), which produces the extrinsic cues conveyed to the laterally adjacent subsidiary mother cells (SMCs). Upon perception of the signal, SMCs become spatially polarized with respect to the GMC, by localized accumulation of cortical F-actin at the SMC/GMC interface and migration of the pre-meiotic SMC nuclei to each interface [4]. The extrinsic cue eventually triggers an asymmetrical division of the SMCs, in an orientation that positions the smaller daughter cell, the SC, adjacent to the GMC [5, 6]. Finally, the GMC produces the two GCs by symmetrical division.

Several intrinsic factors that specify cell fate during the development of GCs have been identified. Moreover, an extrinsic signaling cascade has been dissected in the control of the number and plane of asymmetric divisions [7]. In *Arabidopsis*, three of basic helix-loop-helix (bHLH) transcription factors, SPEECHLESS (SPCH), MUTE and FAMA sequentially modulate the initiation, transition and differentiation events, respectively, regulating the cell lineage involved in the production of stomata [8–13]. The earliest acting bHLH protein, SPCH, functions in the transition from protoderm cells (PDCs) to meristemoid mother cells (MMCs) [10, 12]. Inhibition of SPCH function predominantly affects MMCs and meristemoids and their ability to divide asymmetrically [9, 10, 14], suggesting that SPCH is necessary for the asymmetric divisions that establish the stomatal lineage. The second bHLH protein, MUTE, which is required to terminate asymmetric division and stimulate transition from meristemoid to GMC identity, upregulates a series of cell-cycle genes to drive symmetric division of stomata [12, 15, 16]. Meristemoids of *mute* mutants undergo excessive amplifying divisions and fail to transition to a GMC [12]. Interruption of cell to cell signaling can instigate an extension or reduction of meristemoid division, consistent with the observation that epidermal cell number, including stomata, is a plastic trait that is monitored and adjusted based on internal and external cues [8, 12, 17, 18]. The third bHLH protein, FAMA, controls the final fate transition from GMC to GC, consisting of two distinguishable events: symmetric cell division of the GMC and GC transition [11].

Recently, stomatal initiation in the grass *Brachypodium distachyon* has been shown to involve the orthologs of *Arabidopsis* stomatal bHLH regulators. However, it appears that the function and behavior of the individual components and their interacting regulatory networks have diverged, acquiring specific functions for the unique stomatal development of grasses [19, 20]. *BdMUTE*, an ortholog of *Arabidopsis MUTE* in *B*. *distachyon*, is expressed in GMCs before moving to neighboring cells, and this mobility is a protein-intrinsic feature [21]. In maize, a leucine-rich receptor-like kinase (LRR-RLKs), PANGLOSS1 (PAN1), has been found to participate in transmission of the signals for establishing polarity in SMCs [22]. About 40% of SMCs in loss-of-function *pan1* mutants had unpolarized nuclei and developed defective stomatal complexes, resulting from abnormal asymmetric cell division and SC patterning [22, 23]. Interestingly, PAN2 is required for the subsequent polarization of PAN1 and the Rho family GTPases (ROPs) [24, 25]. After PAN2 polarization, PAN1 and ROP2/9 are polarized, an actin patch forms at the SMC/GMC contact sites, and the SMC nuclei migrate to this site in an actin-dependent manner. *pan2*, *pan1*, *rop2* and *rop9* mutants have abnormal SCs, and their SMCs are defective in actin patch formation and nuclear migration [26, 27]. It has been proposed that the GMC may send a cue to the neighboring cells inducing the asymmetric cell division of the SMCs at adjacent sites to produce the SCs [21, 22]. However, the molecular mechanisms by which GMCs control polarization and cell fate of SMCs in maize remain unclear.

Here we identify *BZU2/ZmMUTE* (*B**i**zu**i2*, abbreviated to *BZU2*) gene, encoding a bHLH transcription factor, an ortholog of *Arabidopsis* MUTE protein. Loss-of-function *BZU2/ZmMUTE* mutants show no SCs and are defective in stomatal complexes. In addition, *bzu2-1* displays defects in symmetric GMC division and lacks SMC polarization. We found that BZU2/ZmMUTE controls the expression of several essential genes involved in the formation of the stomatal complex, and binds to the promoters of *PAN1* and *PAN2* for amplifying the expression of the polarity program in SMCs. Our finding suggests that BZU2/ZmMUTE functions as an important player both acting to regulate the GMC to GC fate transition, and serving as an intercellular signal required for polarization and recruitment of the SMCs.

## Results

### Isolation of *BZU2* mutants showing aberrant stomatal development and defects in water transpiration

To isolate stomatal response deficient mutants, we produced an extensive collection of mutagenized maize lines. Pollen from the maize inbred line Mo17 was treated with ethyl methane sulfonate (EMS) and the M1 progeny were self-pollinated to produce M2 mutant seeds. We screened ~45,000 of these M2 at the seedling stage for drought-sensitivity, using a far infrared thermal imaging approach [28, 29]. We identified a number of mutants displaying an unusually high leaf-surface temperature and designated these as *b**i**zu**i* which means *closed mouth* (abbreviated to *bzu*).

**Fig 1:**
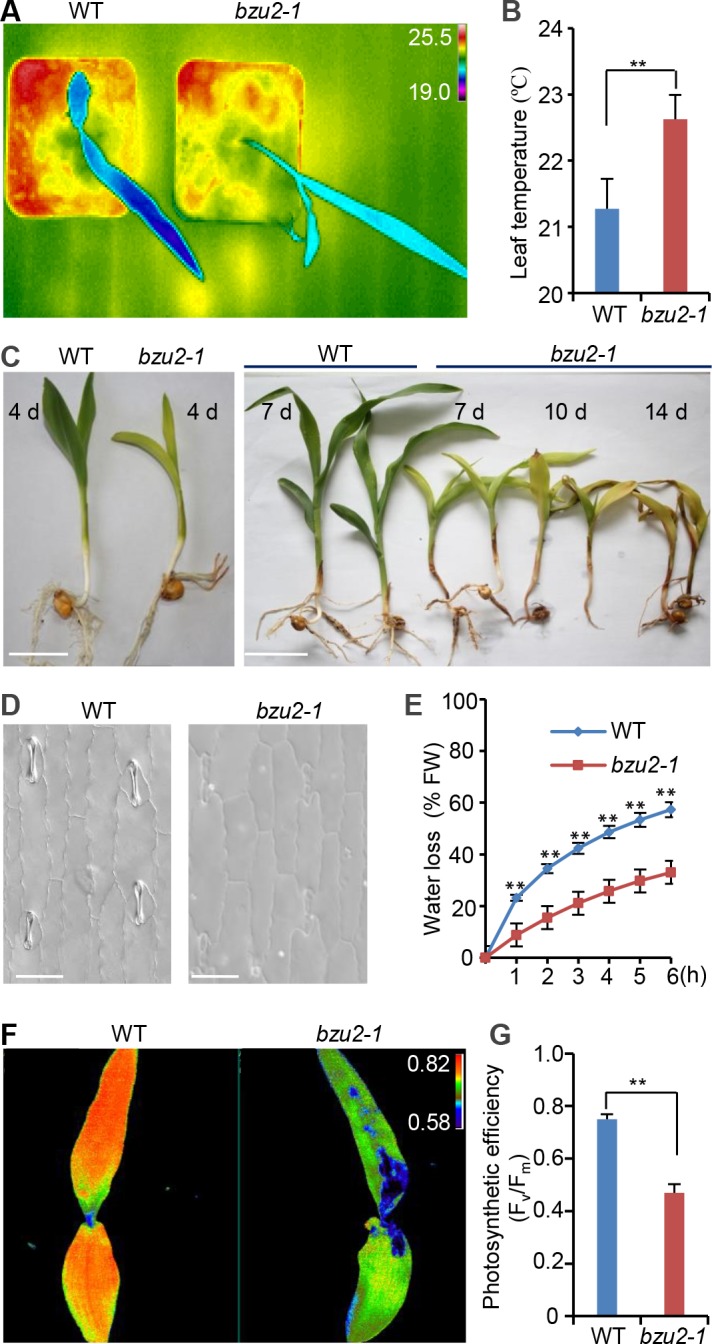
*BZU2* (*Bizui2*) is required for the formation and development of guard cells and subsidiary cells. (A) False-color infrared image of 8-day-old wild-type and *bzu2-1* mutant plants grown under unstressed conditions in soil. The temperatures (°C) of the first and second leaf surfaces are pseudo color-coded according to the scale (inset). (B) Quantitative temperature measures of wild-type and *bzu2-1* leaves. Error bars indicate SD, n = 10, **P<0.01, Student’s *t* test. (C) Morphological phenotypes of *bzu2-1* mutant seedlings. 4-day-old *bzu2-1* and wild-type plants grown in soil. Seedlings of *bzu2-1* mutants show a pale and hyper-hydration phenotype (right panel, scale bar, 1.0 cm). Phenotypes of wild-type and *bzu2-1* seedlings 7, 10 and 14 days after germination illustrating the progressive deterioration of *bzu2-1* seedlings (right panel, scale bar, 1.0 cm). Images are representative results from three independent experiments. (D) Images of the stomatal phenotypes of wild-type and *bzu2-1* mutants. Differential interference contrast (DIC) images were taken from the first leaf of 5-day-old seedlings grown in a growth chamber (16 h/8 h light/dark cycle at 26–30°C). Scale bars, 50 μm. (E) Transpirational water loss in *bzu2-1* mutants is significantly lower than wild-type. 8-day-old leaves of whole seedlings were detached and allowed to dry at room temperature for the indicated periods. Water loss is presented as a percentage of the initial fresh weight (FW) of the seedlings, and results were collected from three independent experiments. Error bars indicate SD, n = 3, **P < 0.01, Student’s *t* test. (F) F_v_/F_m_ images of the first and second leaves of wild-type and *bzu2-1* mutant plants grown under unstressed conditions in soil. The chlorophyll fluorescence images were collected from 8-day-old seedlings. The intensity of F_v_/F_m_ ratio was pseudo color-coded according to the scale. (G) F_v_/F_m_ quantification of the fluorescence from first and second leaves of 8-day-old wild-type and *bzu2-1* mutant seedlings. Each value represents the mean (± SD) of three independent experiments. Error bars indicate SD, n = 6, **P < 0.01, Student’s *t* test.

One of these recessive mutants, *viz*. *bzu2-1*, was studied in detail. It constitutively displays a hot leaf surface phenotype as compared to the wild-type (Mo17) (Fig 1A). In addition, the average leaf temperature of *bzu2-1* mutants was ~1°C higher than wild-type, which reflects an inability of *bzu2-1* leaves to appropriately regulate transpirational water loss from their surfaces (Fig 1B). The *bzu2-1* mutant produced pale, highly hydrated, translucent leaves, and the seedlings died about 14 days after germination (Fig 1C). Upon examination of leaf structure, we observed that the normal mature four-celled stomatal complexes, comprising two dumb-bell shaped GCs adjacent to two SCs, were absent from *bzu2-1* mutants (Fig 1D). As a consequence, the water loss from the leaf surfaces of *bzu2-1* mutants was significantly slower than that of the wild-type (Fig 1E). We subsequently characterized chlorophyll fluorescence emission, comparing the maximum quantum yield (F_v_/F_m_) of wild-type and mutant plants. We found that leaves of the *bzu2-1* mutant have a lower F_v_/F_m_ value than that of wild-type (Fig 1F and 1G), which implies that the photosynthetic activity is dramatically decreased in 8-day-old seedlings of the *bzu2-1* mutant. These observations indicate that the *bzu2* mutant lacks the normal physiological functions of stomata, and is consistent with the lethal seedling phenotype.

### *bzu2* mutants have defects in symmetric GMCs divisions, and lack SMCs

**Fig 2:**
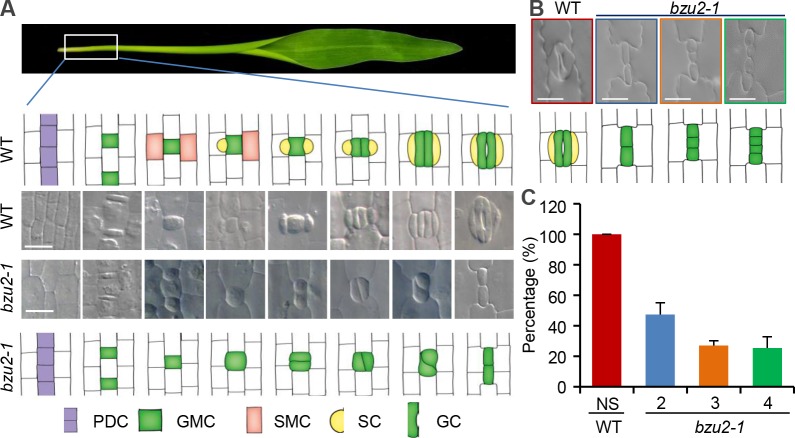
*BZU2* is required for the formation of guard cells and fate determination of subsidiary mother cells. (A) Diagrams show the stomatal development file of the base of the second leaf, comparing wild-type and *bzu2-1* mutants. In maize leaves, stomata are arranged linearly, being strongly zoned with the youngest at the base of the leaf (first panel). The cell proliferation zone of the files (purple region), where the stomata will form, is established first. Next, an asymmetric division of the protodermal cell establishes a GMC (green) and laterally induces SMC fate (orange). Subsequently, SMCs divide asymmetrically to form the SCs (yellow), prior to a final symmetric division of the GMCs to form the mature stomatal complex, comprising two GCs (green) and two SCs (yellow) (second and third panel). Compared to the wild-type, in *bzu2-1* mutants, PDC can develop to GMCs at early stages of this process. However, SMCs do not undergo the asymmetric division to form the SCs, and the longitudinal symmetric division of GMCs is disturbed. Instead, they undergo transverse or diagonal longitudinal divisions that produce short columns of elongated cells lacking characteristic guard cell morphology (fourth and fifth panel). Scale bars, 25 μm. (B) Images of stomata illustrating the phenotypic range observed in *bzu2-1* mutants in the first leaf. Wild-type plants have 4-celled stomatal complexes comprising two GCs and two SCs. *bzu2-1* mutant plants never make SCs and, instead, produce two-celled defective stomata. These sometimes undergo one or two more divisions to form 3- and 4-celled defective stomata. Scale bars, 25 μm. (C) Percentage of stomatal complexes showing irregular divisions of the GMCs in *bzu2-1* mutants. In the first leaves from 8-day-old wild-type and *bzu2-1* mutants, three independent micrographs were examined, each value representing the mean (± SD), with error bars indicating SD, n = 6, **P<0.01, Student’s *t* test. In wild-type plants, all stomata are normal (NS, 100%). In comparison, no normal stomata (NS, 0%) were seen in *bzu2-1*. Out of 252 stomata surveyed in *bzu2-1* mutants, 48% contained 2 cells, 27% contained 3 cells, and 25% contained 4 cells. Abbreviations: Normal stomata (NS), two-celled (2), three-celled (3) and four-celled (4).

As illustrated in Fig 2A, in the wild-type, protodermal cells (PDC) undergo an asymmetric division producing a smaller daughter cell, which is the precursor of an apical GMC, and a larger basal interstomatal cell. Following the differentiation and elongation of the GMCs, the two flanking cells acquire SMCs identities prior to the formation of the GCs. The SMCs then undergo another asymmetric division, producing the two SCs prior to a final division of the GMC, which divides symmetrically and longitudinally to produce the two GCs. The stomatal complexes with dumbbell-shaped GCs immediately adjacent to two triangular SCs are linearly separated by rectangular interstomatal cells.

In *bzu2-1* mutants, the early phase of stomatal development seems to be normal, with PDCs being able to produce GMCs. However, the longitudinal symmetric division of GMCs is altered in the *bzu2-1* mutants, with asymmetric or irregular directional divisions occurred (a transverse or diagonal longitudinal division), resulting in the production of short columns of elongated cells, which lack hallmarks of GC morphology (Fig 2A). Subsequently, none of GMCs develop into normal stomatal complexes (Fig 2B). Instead, in the *bzu2-1* mutant, 48%, 27% and 25% of GMCs were further divided into 2-, 3- or 4-cell groups of undifferentiated cells, respectively (Fig 2C). Our data imply that one function of BZU2 may be to suppress ectopic or premature guard mother cell divisions.

Interestingly, in *bzu2-1* mutants, GMCs also failed to induce flanking cells to form SMCs, resulting in an almost complete absence of SCs. Examination of 802 stomata in *bzu2-1* mutants showed that ~95% of the abnormal GCs had no SCs, and ~5% remaining had one single abnormal SC (S1 Fig). The lack of SCs suggests that absence of *BZU2* may abrogate the acquisition of cell fate of SMC precursors adjacent to GMCs (Fig 2A and S1 Fig).

### *BZU2* mutation results in the loss of SMC polarization

**Fig 3:**
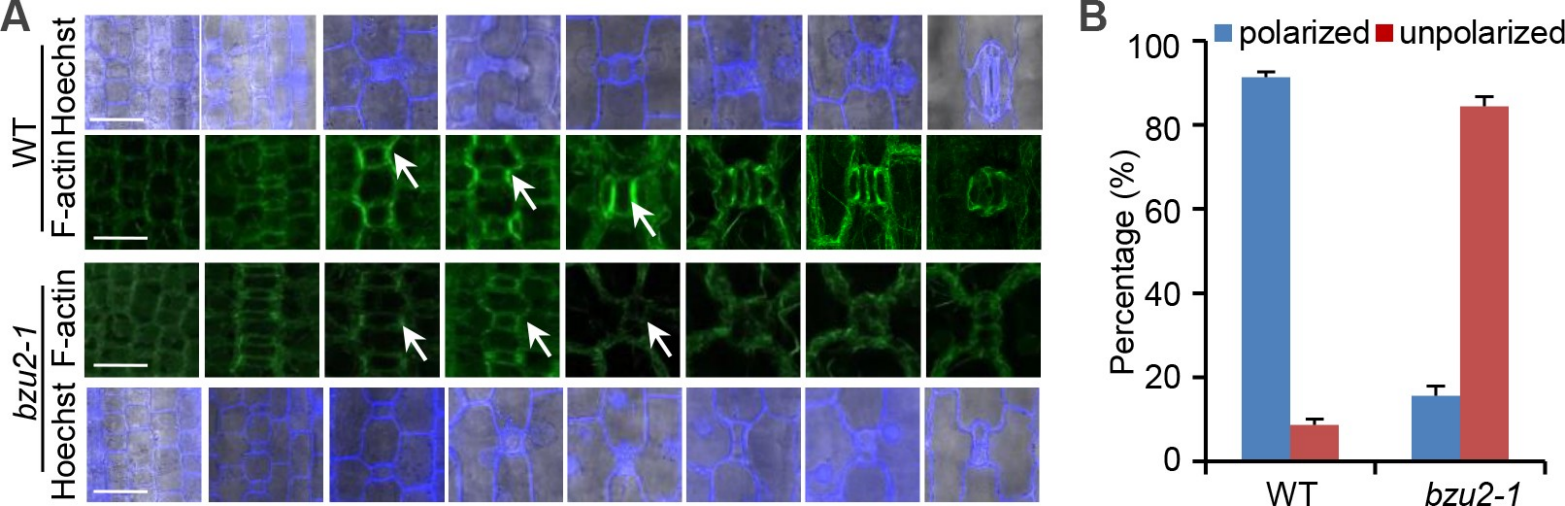
*BZU2* is required for the polarization of subsidiary mother cells. (A) Confocal images of Hoechst 33258-stained (blue, top and bottom panel rows) and F-actin enrichment (green, two panel rows in the middle) in wild-type and *bzu2-1* mutants at the base of the second and third leaves. In wild-type leaves, F-actin enrichment is observed at the SMC/GMC interface (arrowheads, second panel), and the SMC nuclei migrate towards the contact surface. In *bzu2-1* mutants, there is no observable enrichment of F-actin patches at the SMCs/GMCs contact surfaces (arrowheads, third panel), and SMC nuclei fail to polarize. In the wild-type, the SMCs nuclei (blue) migrate to the GMCs (first panel), and in *bzu2-1* mutants, most of the migration of the SMC nuclei (blue) vanishes (fourth panel). Scale bars, 25 μm. (B) Quantitative analysis of SMCs polarization in wild-type (127 SMC nuclei) and *bzu2-1* mutants (167 SMC nuclei) at the base of the second and third leaves, for three independent experiments. In the wild-type, 91.3% of the 127 SMC nuclei that were examined migrate to the SMC/GMC contact surface (polarized). In contrast, 84.4% of the corresponding nuclei examined in *bzu2-1* mutants remained unpolarized. Error bars indicate SD, **P<0.01, Student’s *t* test.

We further examined the spatial interactions between the GMCs and SMCs during stomatal development via light and fluorescence microscopy. In the wild-type, GMCs induce the formation of SMCs, which exhibit polarity as defined by the anisotropic positioning of the SMC nucleus with respect to the SMC/GMC contact surface. In this process, an important step in SMCs polarization is the formation of a dense patch of F-actin, which presumably mediates nuclear migration or anchoring during cell division [22]. In the wild-type, when actin-patches appeared at the SMC/GMC contact sites, the SMCs nucleus will migrate to the contact sites, and then the SMCs undergo one asymmetric division to form SCs (Fig 3A). Our data demonstrated that 91.3% of the SMCs nuclei migrated to the SMC/GMC contact surface in the wild-type, the cells therefore being polarized and highly anisotropic. In contrast, 84.4% of the SMCs examined in *bzu2-1* mutants were non-polarized, with the nucleus being centrally located (Fig 3A and 3B). These results show that BZU2 either acts as an intrinsic signal in SMC development, or controls the expression of intrinsic factors that determine SMC polarity and, ultimately, cell fate.

### Map-based cloning and identification of *BZU2*

**Fig 4:**
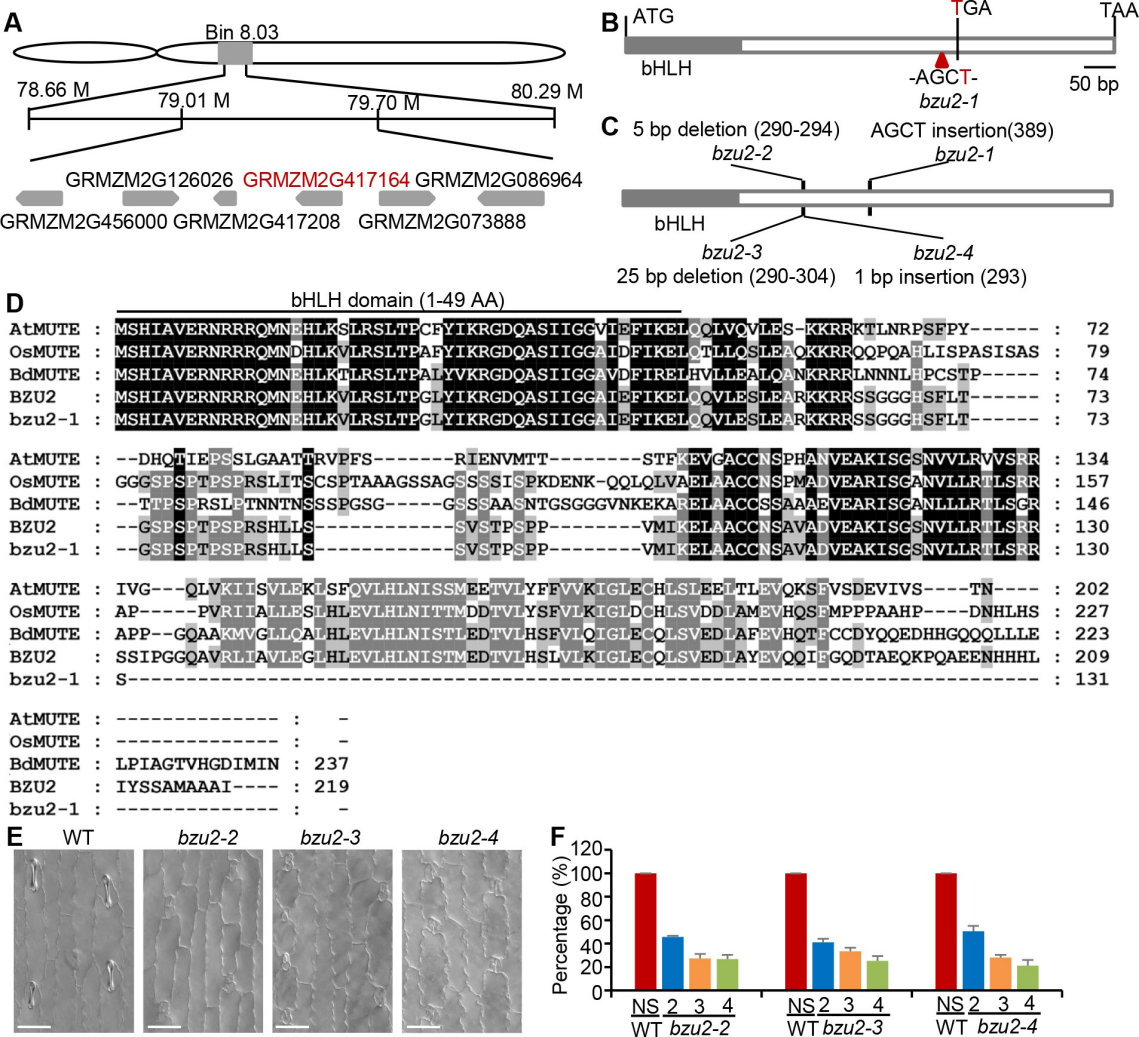
A mutation in *BZU2* is responsible for stomatal defects in maize. (A) *BZU2* was mapped to bin 8.03 of chromosome VIII, between the 79M15 and 79M45 SSR markers, a 0.69 Mb region containing fifteen genes (the key gene is labeled in red). (B) The *bzu2-1* mutation was generated by a 4 nucleotide AGCT insertion at position + 390 of GRMZM2G417164, which generated a stop codon within the reading frame. (C) Structure of *bzu2-1* and the CRISPR/Cas9-induced mutations (*bzu2-2*, *bzu2-3* and *bzu2-4*). *bzu2-2* contains a 5 bp deletion downstream of the PAM site; *bzu2-3* contains a 25 bp deletion downstream of the PAM site; *bzu2-4* contains a 1 bp insertion downstream of the PAM site. (D) Alignment of BZU2 (GRMZM2G417164) (in wild-type and *bzu2-1*), AtMUTE (At3g06120), OsMUTE (LOC_Os05g51820) and BdMUTE (Bradi1g18400). (E) DIC images show similar aberrant GCs phenotypes, as well as a lack of SCs in *bzu2-2*, *bzu2-3* and *bzu2-4* mutants in the first and second leaves. Scale bars, 50 μm. (F) Percentage of stomatal complexes showing irregular GMC divisions in CRISPR/Cas9-induced mutations (*bzu2-2*, *bzu2-3*, and *bzu2-4*) in the T0 generation, based on examination of the first leaves of three 8-day-old wild-type and CRISPR/Cas9 mutants. In wild-type plants, all stomata are normal (NS, 100%). No normal stomata (NS, 0%) were observed in *bzu2-2* (173 stomatal complexes examined), *bzu2-3* (168 stomatal complexes), and *bzu2-4* (150 stomatal complexes). Of 493 stomata surveyed, 48% comprised 2 cells, 28% comprised 3 cells, and 24% comprised 4 cells. Abbreviations: AA: amino acid, Normal stomata (NS), two-celled (2), three-celled (3), four-celled (4).

In order to characterize *BZU2* at the molecular level, we crossed the *bzu2-1* mutant (carried in the Mo17 background) to the inbred line B73, thereby generating reciprocal F1 hybrid progeny. The F2 population resulting from F1 self-crossing was screened based on the *bzu2-1* mutant phenotype (S2 Fig). Initial mapping indicated that the *BZU2* locus co-segregated with the simple sequence repeat (SSR) markers bnlg1863 (recombination rate 2.1%) and umc1858 (recombination rate 12.5%) in Bin 8.03 of chromosome VIII. Several rounds of fine-mapping narrowed this locus to a 0.69 Mb region between SSR markers 79M15 (79.01 M) and 79M45 (79.70 M) containing fifteen genes (Fig 4A). After sequencing, we identified the *BZU2* gene as GRMZM2G417164 (Fig 4B), which encodes a bHLH transcription factor exhibiting sequence similarity to *Arabidopsis*, rice and *Brachypodium* MUTE (Fig 4D and S3 Fig). *bzu2-1* contains a 4 nucleotide ‘AGCT’ insertion at the position 390 bp downstream of the start of transcription generating a premature STOP codon (Fig 4B and 4C). The *bzu2-1* mutant phenotype was further confirmed by targeted gene knockouts of *BZU2/ZmMUTE* using the CRISPR/Cas9 system. Stomatal phenotypes of three independent CRISPR/Cas9-mutated lines (*bzu2-2*, *bzu2-3* and *bzu2-4*) are identical to *bzu2-1*, confirming *BZU2/ZmMUTE* as GRMZM2G417164 (Fig 4E and 4F and S4 Fig). When a *GFP- BZU2/ZmMUTE* fusion chimera was transiently expressed in tobacco (*Nicotiana tabacum* L.), we were able to show accumulation of GFP-BZU2/ZmMUTE protein within the nucleus (S5 Fig), which is consistent with the putative function of BZU2/ZmMUTE as a transcription factor.

### BZU2/ZmMUTE can move from GMC to SMC

**Fig 5:**
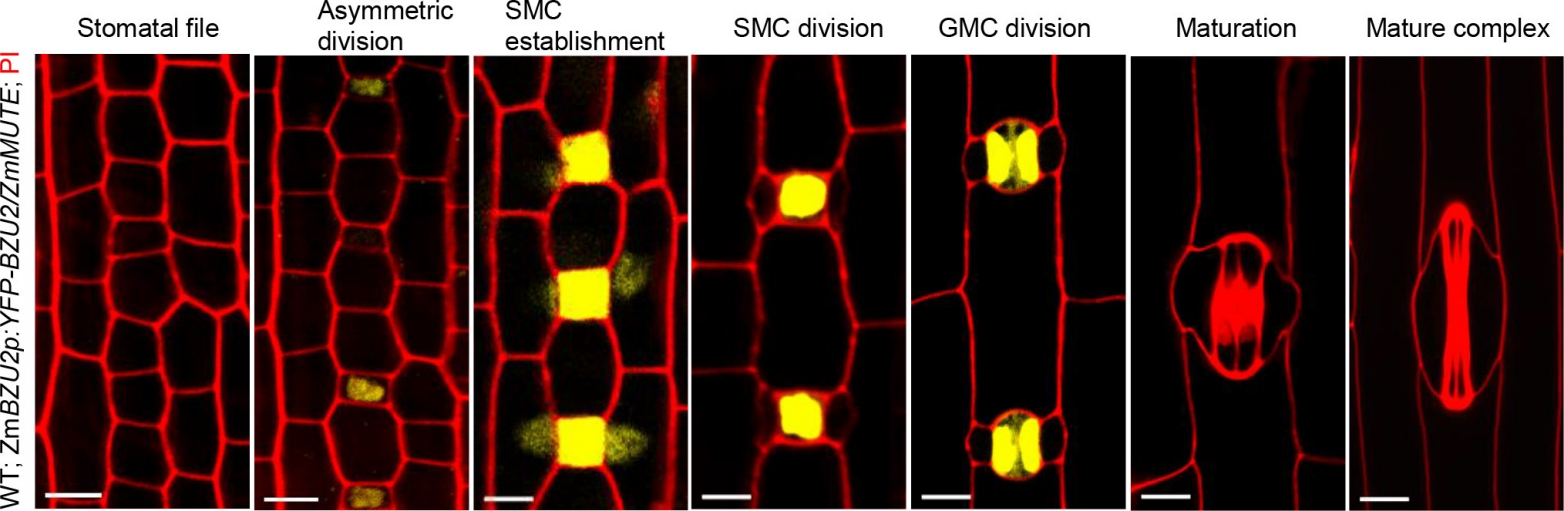
BZU2/ZmMUTE is specifically expressed in the guard mother cell and the subsidiary mother cell establishment stage during stomatal development in maize. *BZU2/ZmMUTEp*:*YFP-BZU2/ZmMUTE* expression in wild type during stomatal development. YFP-BZU2/ZmMUTE is expressed from young GMCs to mature GMCs, and demonstrates a particularly strong signal in mature GMCs and at the SMC establishment stage. Expression of YFP-BZU2/ZmMUTE disappears during stomatal complex maturation after GMC division. These images were observed using the second or third leaf, 4 to 5 dag, of the T1 generation. Propidium iodide (red) was employed to counterstain the cell walls. Scale bars, 10 μm. dag, days after germination.

The interesting role of BZU2/ZmMUTE in controlling neighbor cell fate prompted us to further investigate the behavior of BZU2/ZmMUTE protein in the early developmental stages of SCs and GCs. In the transgenic reporter plants, the fluorescence of *ZmMUTEp*:*YFP-ZmMUTE* was first detected during the stomatal development of early GMCs, and then moved to SMCs in the SMC establishment stage. The YFP-ZmMUTE signal remains strong until the young GCs become mature GCs (Fig 5). Unexpectedly, as compared to the expression pattern of BdMUTE, BZU2/ZmMUTE is more specifically expressed in the SMC establishment stage (Fig 5).

To further test the capability of ZmMUTE to move, YFP-fused BZU2/ZmMUTE, as well as its homologues, BdMUTE, OsMUTE, and AtMUTE, were expressed in rice (*Oryza sativa* L.). *OsMUTEp*:*nls-YFP* was only expressed in the GMC at the developmental stages leading from GMC formation to SMC division (S6A Fig), indicating that *OsMUTE* promoter is active during development of GMC and SMC [13]. Interestingly, as for *OsMUTEp*:*YFP-BdMUTE*, *OsMUTEp*:*YFP-OsMUTE* and *OsMUTEp*:*YFP-ZmMUTE*, the fluorescence of the YFP-fused MUTEs were first detected in the early GMCs, then appeared in the SMCs (S6B–S6D Fig, white arrows). Similar to *ZmMUTEp*:*YFP-ZmMUTE* (Fig 5), after the division of the GMCs, the signals of *OsMUTEp*:*YFP-ZmMUTE* were also observed in young GCs and SCs, finally disappearing from the GCs and SCs at maturity (S6C Fig). In contrast, the fluorescence of *OsMUTEp*:*YFP-AtMUTE* was very weak in the early GMCs and not detected in SMCs (S6E Fig).

**Fig 6:**
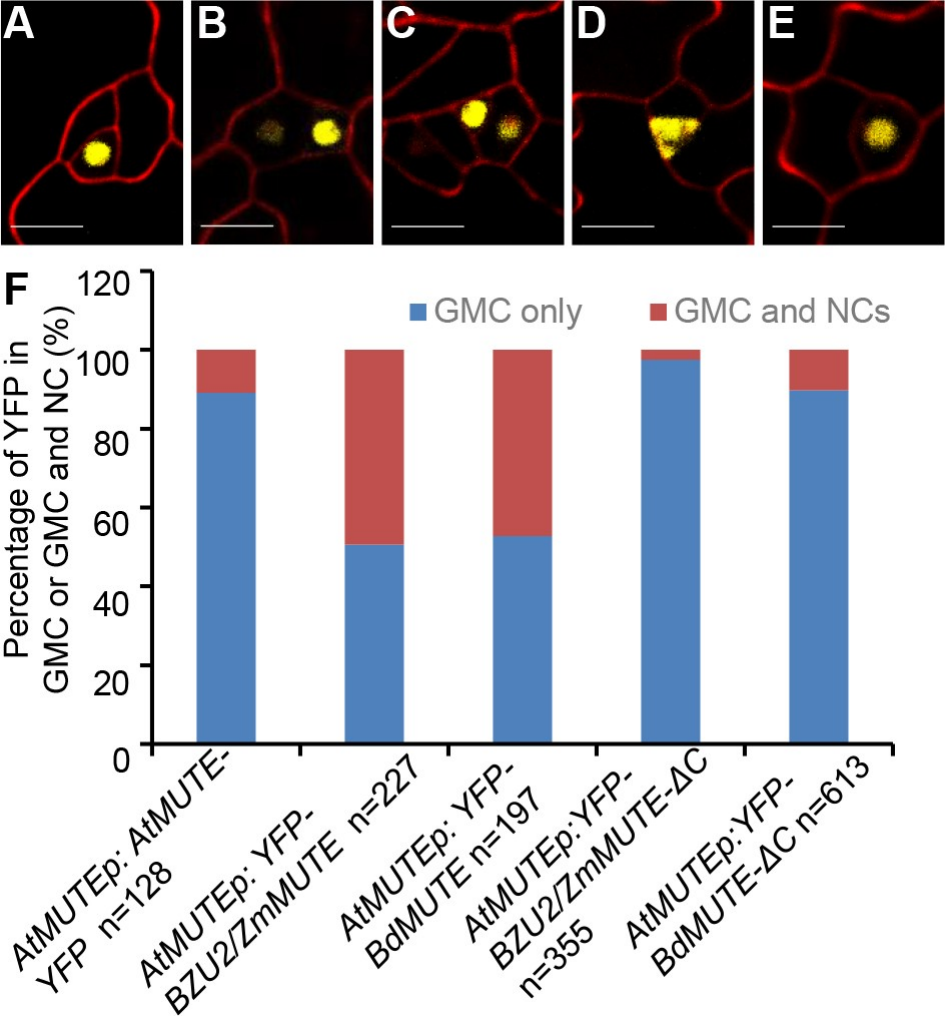
BZU2/ZmMUTE can move from GMCs to neighboring cells in *Arabidopsis*. (A-C) *AtMUTEp*:*AtMUTE-YFP* is exclusively expressed in Guard Mother Cells (GMCs). Expression of *AtMUTEp*:*YFP-BZU2/ZmMUTE* and *AtMUTEp*:*YFP-BdMUTE* is seen in GMCs and neighboring cells. (D-E) The fluorescence of *AtMUTEp*:*YFP-BZU2/ZmMUTE-*ΔC is only observed in GMCs, being located both in the nucleus and the cytoplasm. The fluorescence from *AtMUTEp*:*YFP-BdMUTE-*ΔC expression is seen mostly in GMCs. (F) Quantification of YFP-AtMUTE (N = 128), YFP-BZU2/ZmMUTE (N = 227), YFP-BdMUTE (N = 197), YFP-BZU2/ZmMUTE-ΔC (N = 355), and YFP-BdMUTE-ΔC (n = 613) expression in GMCs and in neighboring cells. *Arabidopsis* images from T2 cotyledons, 3.5 dag. Cell walls are counterstained with propidium iodide (red). Scale bars, 10 μm. dag, days after germination.

Meanwhile, we compared the expression patterns of these different YFP-fused MUTE coding sequences driven by the *AtMUTE* promoter in *Arabidopsis*. *AtMUTEp*:*AtMUTE-YFP* was observed exclusively in *Arabidopsis* GMCs (Fig 6A and 6F), but *AtMUTEp*:*YFP-ZmMUTE* and *AtMUTEp*:*YFP-BdMUTE* were observed both in GMCs and in neighboring cells (Fig 6B, 6C and 6F). Since divergent functions of MUTE have been reported in different species [10, 21], we performed sequence alignments, the results of which indicate that MUTE proteins from different species have a variable C-terminal region, whereas the N-terminal region is relatively conserved (Fig 4D). To further verify the characterization of the C-terminal regions of BZU2/ZmMUTE and BdMUTE, we generated *AtMUTEp*:*YFP-ZmMUTE-ΔC* and *AtMUTEp*:*YFP-BdMUTE-ΔC* transgenic plants in *Arabidopsis* which lack the 190–219 and 208–237 amino acids in ZmMUTE and BdMUTE, respectively. The fluorescence of *AtMUTEp*:*YFP-ZmMUTE*-ΔC is only detected in the GMCs and expressed in both nucleus and cytoplasm. In contrast, *AtMUTEp*:*YFP- BdMUTE*-ΔC is detected in the GMCs and restricted in nucleus, which is similar to the pattern seen with *AtMUTEp*:*AtMUTE-YFP* (Fig 6D–6F). At the same time, we checked the expression patterns of *OsMUTEp*:*YFP-ZmMUTE-ΔC* and *OsMUTEp*:*YFP-BdMUTE*-*ΔC* in rice. As shown in S6F and S6G Fig, *YFP-ZmMUTE*-ΔC and *YFP-BdMUTE*-ΔC are only located in the early GMCs, and not in the SMCs and young SCs.

However, it is obvious that the localization (for ZmMUTE-ΔC, Fig 6D and S6F Fig), or the intensity (for BdMUTE-ΔC, Fig 6E and S6G Fig), does not correspond to that of the wild-type, suggesting that the protein does not properly interact with its binding partner for normal localization and/or stability. To further confirm whether a truncated BZU2/ZmMUTE protein, from which the 30 amino acids of the C-terminus had been deleted, can still bind the E-box motifs within the promoters of *PAN1* (-27) and *PAN2* (-187, -200), the yeast one-hybrid assay and electrophoretic mobility shift assays (EMSA) were used to test the activity of ZmMUTE-ΔC binding to short nucleotide fragments (26–37 bp) containing the E-box motifs. The results of both assays show that BZU2/ZmMUTE lacking these C-terminal amino acids is similar in behavior to BZU2/ZmMUTE (S7 Fig and S8 Fig). These data imply that the C terminus of BZU2/ZmMUTE and BdMUTE might be necessary for their characteristic mobility. Together, these results support that BZU2/ZmMUTE is mobile and necessary for SMC formation and asymmetric division in normal development of the maize stomatal complex.

### BZU2/ZmMUTE modulates gene expression related to GC formation and SC precursor initiation

**Fig 7:**
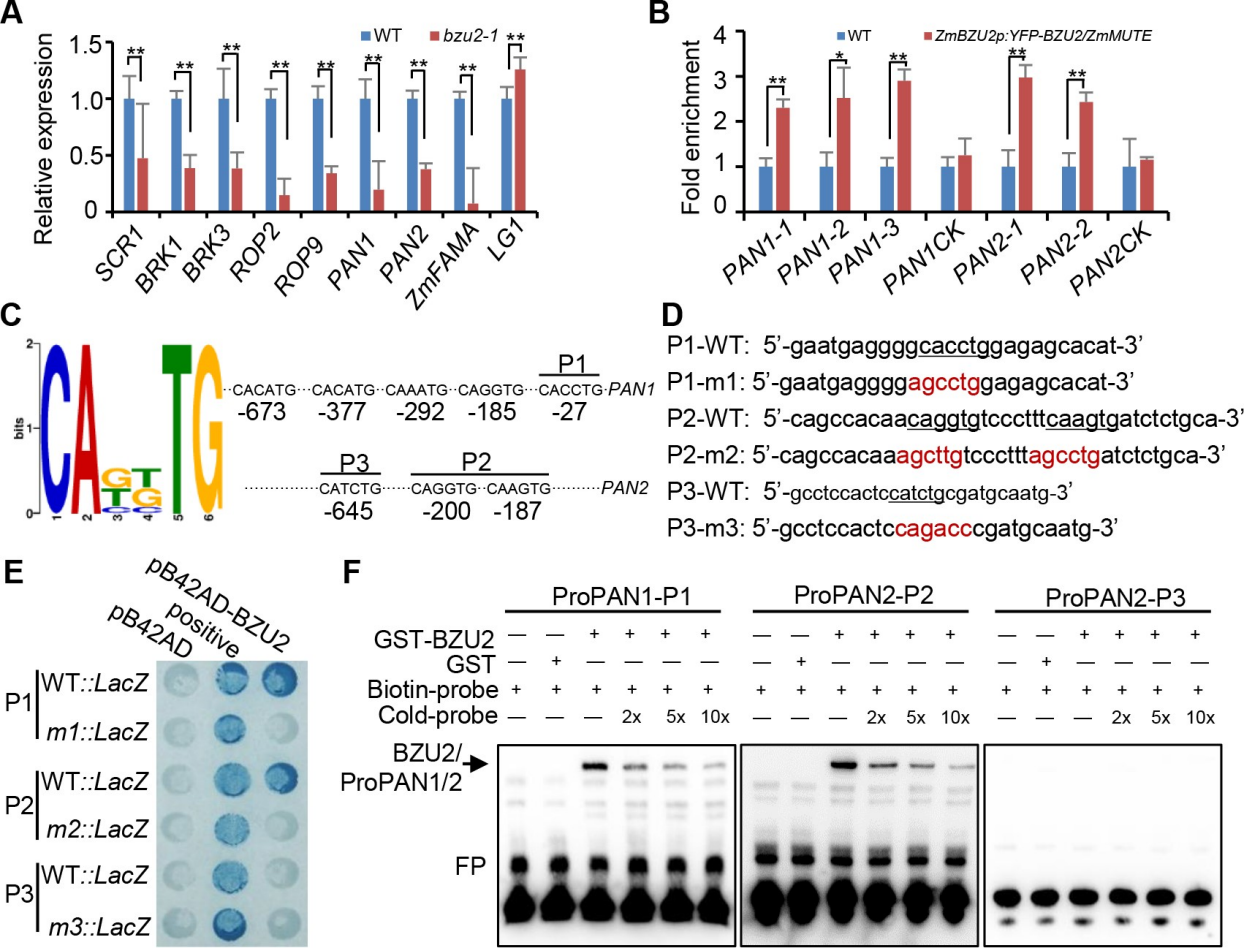
BZU2/ZmMUTE binds to the E-box motifs located in the *PAN1* and *PAN2* promoters. (A) RT-qPCR analysis of the expression of the genes associated with stomatal development indicates repression of transcription in *bzu2-1* mutant plants. RNA was taken from samples excised up to ~1.5 cm from the base of second and third leaves. RT-qPCR values are expressed as the mean ± SD compared to that of the internal control (*ZmUbiquitin 2*). Error bars indicate SD, n = 3, Student’s *t* test, **P < 0.01. Assays were done in triplicate. (B) ChIP-qPCR results indicating that the promoter fragments of *PAN1*and *PAN2*, can be amplified from the immunoprecipitates pulled down by the anti-GFP antibody. The sequences used for ChIP-qPCR contain the E-box *cis*-elements located within the *PAN1* and *PAN2* promoters. Second and third leaf base sections (~1.5 cm) of 8-day-old seedlings were harvested for the chromatin immunoprecipitation (ChIP) experiments. Sequence from regions of the *PAN1* and *PAN2* promoters lacking the E-box *cis*-elements were used as controls. Error bars indicate SD, n = 3, Student’s *t* test, **P<0.01. (C) The E-box motifs in the promoters of *PAN1* and *PAN2*. The bHLH transcription factor binding the *cis*-element consensus sequence (E-box) and their locations in the promoters of *PAN1* and *PAN2* (-750 - -1). The E-box (6 bp) motif logo was produced by MEME based on previously studied motifs. The relative position of E-box *cis*-elements in the promoters of *PAN1* and *PAN2* as detected by ChIP-qPCR. (D) The DNA fragments used for yeast one-hybrid and EMSA assays. The E-box is underlined, and the mutated sequences are highlighted in red. (E) Yeast one-hybrid assays showing BZU2/ZmMUTE binding to the E-box motifs present in the *PAN1* and *PAN2* promoters. (F) EMSA assays showing specific binding of BZU2/ZmMUTE to the E-box motifs in the *PAN1* and *PAN2* promoters. EMSA was performed with probes that were biotin-labeled (biotin probe) or unlabeled (cold probe). E-box-containing DNA fragments (top panel) and recombinant BZU2/ZmMUTE protein; specific combinations are shown above the autoradiograph. Unlabeled fragments were added gradually in 2-, 5- or 10- fold excess, as indicated.

In order to further assess the role of BZU2/ZmMUTE in stomatal development and, in particular, define its interactions with other cellular components, we performed RT-qPCR to compare, for *bzu2-1* mutants and wild-type, the transcript levels of genes previously reported to be involved in stomatal and leaf development (Fig 7A). PAN2 is polarized in premitotic SMCs [24]. After PAN2 polarization, PAN1 and ROP proteins are polarized, and an actin patch forms at the GMC/SMC interface [22, 23]. ROP2/9 functions downstream of PAN1 to promote the premitotic polarization of SMCs [25], and the premitotic SMC nucleus migrates to this site in an actin-dependent manner. *Liguleless1* (*LG1*) accumulation at the site of ligule formation and in the axil of developing tassel branches, functions in the leaf shape and tassel architecture [30]. Low levels of the *PAN1*, *PAN2* and *ROP2/9* transcripts were observed in *bzu2-1* mutant plants as compared to wild-type. Furthermore, these genes related to stomatal development are down-regulated at very early seedling stages (S9 Fig), since stomata production in grass initiates at the leaf base with a longitudinal gradient of development and differentiation toward the tip. This therefore suggests that the reduction of the transcript levels of these genes is not simply due to the leaves dying. For example, *ROP2* transcript abundance in the wild-type was 6.7 times higher than that in *bzu2-1*. Transcripts of the *Brick1* (*BRK1*) and *BRK3* genes, required for the formation of epidermal cell lobes as well as for actin-dependent cell polarization events of subsidiary mother cells [26, 31, 32], were also found at lower levels in *bzu2-1* plants. The expression of *SCARECROW* (*SCR*) gene in rice and maize was observed in leaf primordia and in young leaves, which is required for asymmetric cell divisions of GMCs [13, 26, 33]. The transcript level of *SCR1* was significantly reduced in *bzu2-1* as compared to wild-type. However, the transcript levels of *LG1*, *a SQUAMOSA PROMOTER-BINDING proteins 1* and *2* like gene, in *bzu2-1* mutants was comparable to that in the wild-type.

More importantly, it is also known that the function of FAMA and OsFAMA is conserved between dicots and monocots in the regulation of the final symmetric division of the GMCs [13, 34]. In rice, *c-osfama* (*OsFAMA* mutant in rice, generated by CRISPR/Cas9) mutants showed the stomatal complex consisted of four swollen cells, two GCs and two SCs, occasionally a stoma lacking one SC was observed, however in *c-osfama* the entry division and GMC differentiation stage even the stomatal density is the same compared with wild-type. OsFAMA controls the cell fate transition from GMCs to GCs and SMCs to SCs and affected SMC asymmetrical division [13]. We noticed that, in *bzu2-1*, the expression of *ZmFAMA* is reduced significantly (Fig 7A), similarly to the manner in which *OsFAMA* is dramatically downregulated in the *c-osmute* mutant [13]. Therefore, the absence of *ZmFAMA* or *OsFAMA* is simply a result of the lineage abortion. These data clearly demonstrate that the genes of asymmetric and symmetric division in *bzu2-1* mutants are impaired in stomatal development, which is consistent with the proposed role of *BZU2/ZmMUTE* as a master regulator of stomatal differentiation. Therefore, it is speculated that the abnormal formation of SCs and the symmetric division of GMCs in *bzu2-1* mutants might be specifically due to downregulation of *PAN1*, *PAN2*, and *ZmFAMA* (Fig 7A and S9 Fig).

Previous studies have shown that bHLH transcription factors can specifically bind to the E-box *cis*-element and regulate the expression of targets (Fig 7C) [35]. Therefore, we performed motif enrichment analysis by MEME [36], found that five and three E-box *cis*-elements are located in the regions within -750 - -1 in the promoter of *PAN1* and *PAN2*, respectively (Fig 7C). This prompted us to assess the DNA binding specificity of BZU2/ZmMUTE. First, we obtained transgenic plants of *BZU2/ZmMUTEp*:*YFP-BZU2/ZmMUTE*, and used for ChIP-qPCR experiments using anti-GFP (ab290, Abcam) polyclonal antibody (the expression of YFP-BZU2/ZmMUTE was confirmed by Western blot using anti-GFP antibody) (S10 Fig). Our results showed a strong enrichment in promoter fragments for *PAN1* and *PAN2*, as compared to negative controls (the wild-type in the presence of anti-GFP). However, no enrichment was observed in the negative control using the regions lacking E-boxes in the promoters of *PAN1* and *PAN2* (Fig 7B). These data are consistent with the ChIP-qPCR results using the native antibody of BZU2/ZmMUTE (S11 Fig). Second, we employed the yeast one-hybrid assay to directly examine interactions between BZU2/ZmMUTE and 3–4 repeats of short nucleotide fragments (26–37 bp) containing the E-box motifs from the promoters of *PAN1* (-27) and *PAN2* (-187, -200). Activation of BZU2/ZmMUTE the *LacZ* reporter gene was seen for *PAN1* sequence motifs, but was not detected using sequences from a second putative E-box contained in the *PAN2* promoter (-645) (Fig 7C–7E). Mutation of the E-box motif eliminated *LacZ* expression, providing confirmation of the binding specificity of BZU2/ZmMUTE. Finally, the experiment of EMSA further indicated that BZU2/ZmMUTE binds to the E-box motifs *in vitro* (Fig 7F), which is consistent with the yeast one-hybrid data. Taken together, our results suggest that *PAN1* and *PAN2* are two direct targets of BZU2/ZmMUTE.

## Discussion

In this study, we found that *BZU2/ZmMUTE* encoding a bHLH transcription factor is an ortholog of AtMUTE. *AtMUTE* plays an essential role in the transition of the meristemoid to GMC by repressing the stem cell activity of the meristemoid and inducing guard mother cell formation [10, 12]. Loss-of-function *bzu2-1* mutants can form normal GMCs, but fail to undergo a symmetric division to generate two guard cells, as in wild-type. This indicates that the GC precursors in *bzu2-1* are similar to wild-type, with the exception of stomatal development. Consequently, eight-day-old seedlings of *bzu2-1* displayed etiolated phenotypes and, at the stage of three leaves, the seedlings subsequently died (Fig 1). Unlike *AtMUTE* mutants, the longitudinal symmetric division of the GMC in *bzu2-1* mutants is replaced by an asymmetric or irregular directional division (transverse or diagonal longitudinal division), resulting in the production of 2–4 short columns of elongated cells (Fig 2B and 2C). In *bzu2-1* mutants, the stomata are defective and thus the functions of gas exchange and water loss are impaired. Our data suggest that BZU2/ZmMUTE acts as a switch that controls stomatal development.

Normally, in maize, premitotic SMCs polarize toward the GMC in response to hypothetical cues coming from the adjacent GMCs. This process involves migration of the nucleus toward the GMCs and a distinct enrichment of cortical F-actin at the point of interaction of the GMCs and SMCs when the cell files are forming. Subsequently, SMCs divide asymmetrically to produce subsidiary cells flanking the GMC, which in turn divides to produce a guard cell pair to form stomatal complex [4]. In *bzu2-1* mutants, a lack of nuclear polarization and/or actin accumulation at the GMC/SMC interaction area was observed. Thus, the GMCs failed to induce the flanking cells to form SMCs. *bzu2-1* mutants share similar features with *BdMUTE* mutants of *B*. *distachyon*, but are different in several respects (Fig 2). Both mutations of *MUTE* in *Zea mays* and *B*. *distachyon* result in an abnormal SMC formation and polarization. *BdMUTE* mutants fail to recruit SCs and instead produce dicot-like two-celled stomata [21]. As compared to *BdMUTE*, where partial guard cells form without SCs, *bzu2-1* is completely defective in guard cell and SMC formation. These data suggest that the function of BZU2/ZmMUTE transcription factor in maize is different from BdMUTE, since absence of the former appears to disrupt SC and GC formation in a more direct and severe manner than does absence of BdMUTE. In addition, our results suggest the C-terminal is necessary for the mobile nature of both BdMUTE and BZU2/ZmMUTE (Fig 6).

Even though previous work in *B*. *distachyon* has established the mobile nature of BdMUTE, the molecular mechanisms of how BdMUTE controls SCs formation are still unknown [21]. In fact, the mechanisms governing SMC polarization to allow establishment of asymmetric division are also largely unknown. We provide several lines of evidence suggesting that BZU2/ZmMUTE participates in the regulation of SMC development, and of PAN1 and PAN2, which are early regulators of SC precursor and of SMC polarization by cooperatively promoting polarization of the actin cytoskeleton and nuclei in these cells [22]. Firstly, our data show that BZU2/ZmMUTE can bind to the E-box of the *PAN1* and *PAN2* promoters (Fig 7 and S7 Fig); consistent with this, transcript levels for *PAN1* and *PAN2* are severely downregulated in *bzu2-1* mutants as compared to the wild-type. Unexpectedly, yeast one-hybrid and EMSA data show that BZU2/ZmMUTE is not able to activate P3 of the promoter of *PAN2 in vitro*, but the ChIP-qPCR data indicate that BZU2/ZmMUTE can activate P3 of the *PAN2* promoter *in vivo*. We speculate that BZU2/ZmMUTE combines with additional unknown factors to activate P3 of the *PAN2* promoter *in vivo* (Fig 7, S7 Fig and S8 Fig), by analogy to the observation that the DNA binding specificity of AtMUTE depends on its dimerization partner ICE1 or SCRM2 [37]. Furthermore, a number of genes involved in the regulation of stomatal development are down-regulated in *bzu2-1* mutants as compared to the wild-type (Fig 7A). Finally, mutations in *BZU2/ZmMUTE* disrupted the actin-based patch attachment of GMCs with SMCs, which can mis-orient the deposition of new cell walls (Fig 3A and 3B) [38–41]. Actin plays an important role in the spatial regulation of asymmetric cell division. For example, actin-dependent relocation of the nucleus during G1 to a defined cortical site of SMCs during stomatal complex formation is one of the early events of asymmetric cell division in *Tradescantia* [42] which is followed by formation of a dense actin patch at this site [43, 44].

**Fig 8:**
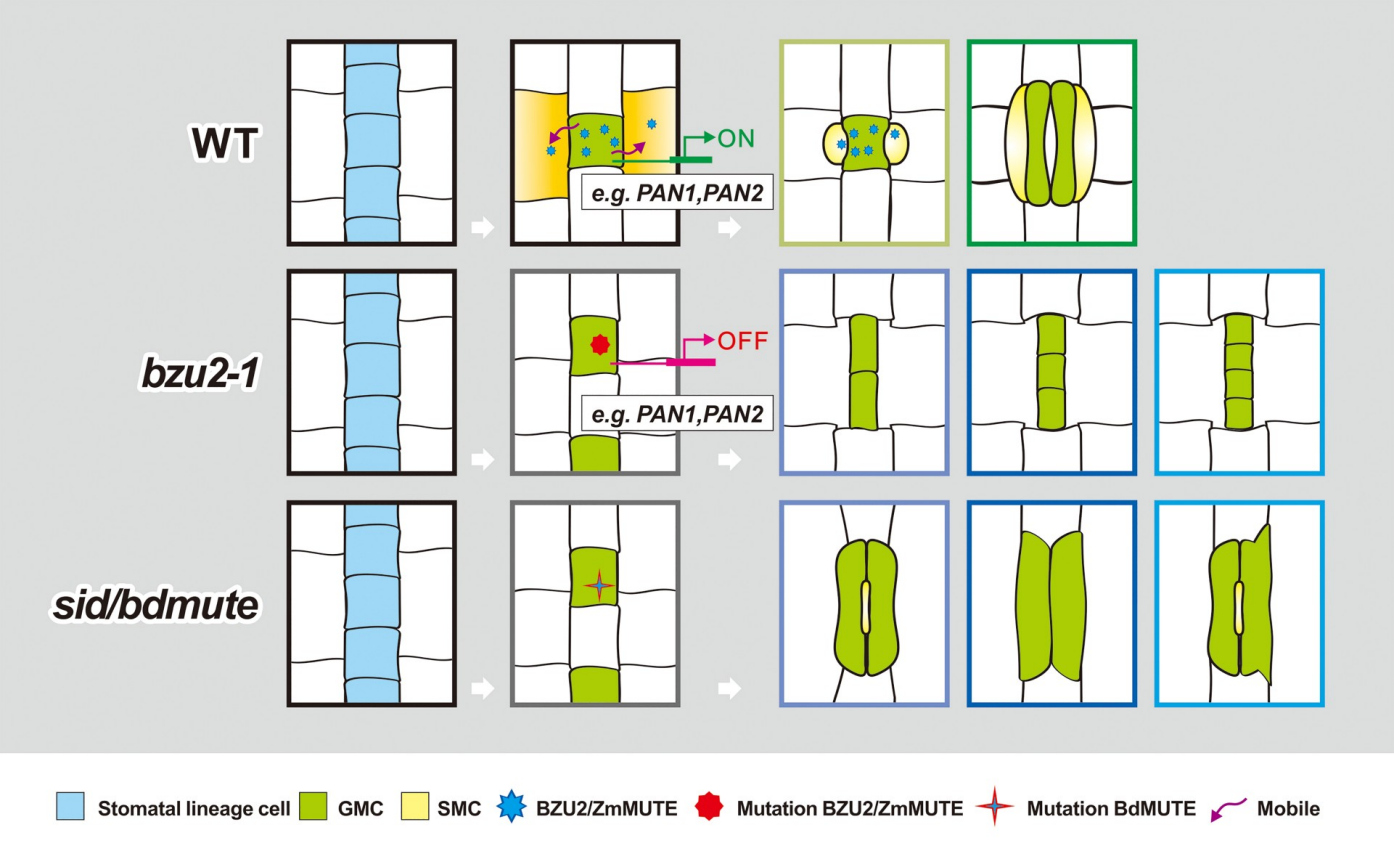
A schematic illustration of a plausible model of stomatal development modulated by *BZU2/ZmMUTE*. The *bzu2-1* mutants show defects in the symmetrical division of GMCs, and also lack nuclear polarization and/or actin accumulation at the SMC/GMC interface. Defects in premitotic SMC polarization precludes the SMCs from forming SCs. SMC: subsidiary mother cell; GMC: guard mother cell; PC: pavement cell; *sid*: *subsidiary cell identity defective*.

Combining the existing knowledge and the results presented, here we propose a model in which BZU2/ZmMUTE plays a role partially similar to what has been previously described, but that includes some different roles in maize stomatal development (Fig 8). In the base of maize leaves, protodermal cells undergo one asymmetric division to form the GMCs. At a specific stage of GMC development (Fig 5), these cells could send BZU2/ZmMUTE as an extrinsic cue to neighboring cells connecting non-sister GMCs and SMCs, thereby acting as an important regulator of the early players in SC development (e.g. *PAN1*, *PAN2*). After BZU2/ZmMUTE-mediated induction, PAN1 and PAN2 (and possibly other additional factors) accumulate at the SMC/GMC interface, working together with F-actin to induce SMC polarity and nuclear migration towards the GMC proximal site [26]. Following polarization, SMCs undergo one asymmetric division to form a SC and an epidermal cell. In the final stage of stomatal development, BZU2/ZmMUTE performs an additional role controlling the symmetric division of the GMCs to produce the two GCs. It is important to note that the role of BZU2/ZmMUTE in the biogenesis of SCs is not limited to the control of *PAN1/2* expression, since SC formation is not entirely disrupted in *pan1* or *pan2* mutants, whereas SCs are completely absent in *bzu2-1* mutants [22]. Thus, these imply that BZU2/ZmMUTE, acting as a GMC-derived polarizing signal, moves to neighboring cells (Fig 5). This, in turn, initiates the expression of the polarity program by regulating the expression of the genes required for nuclear polarization and polarized actin accumulation at the GMC contact sites (Fig 7A). These data indicate that BZU2/ZmMUTE may play a role in the modulation of gene expression at earlier stages of SC precursor development. In summary, our data support a critical role for BZU2/ZmMUTE in the regulation of SC development and GC maturation. Further studies will be required to dissect the functions of BZU2/ZmMUTE in the determination of GMC fate and in the initiation of intercellular signaling required for the recruitment of the SMCs during stomatal development. More insights could emerge from characterization of the additional mutants obtained in our screen that show different defects in GC and SC development.

## Materials and methods

### Plant materials

Maize plants were grown at the experimental station of Henan University in the Kaifeng experimental field, Henan Province, and the Sanya experimental field, Hainan Province.

### Collection of EMS-mutagenized plants

To isolate stomatal development deficient mutants, we established an extensive collection of EMS-mutagenized maize plants, as follows: the pollen of maize inbred line Mo17 was treated with EMS, T1 population seeds were sowed in the soil, and T1 plants were self-crossed to obtain T2 population seeds. We screened the T2 mutagenized population for the phenotype of an altered leaf surface temperature, using a far-infrared imaging instrument. In 3,000 lines, represented by ~45,000 T2 population seeds, we found a single lethal mutant, and named *bzu2-1* (called *bizui*, *closed mouth*, *bzu2-1*). Genetic analysis indicates *BZU2* is a qualitative trait gene, with a segregation ratio of 3:1 in the F2 generation (Table 1).

**Table 1:** x^2^ goodness-of-fit tests for *bzu2-1* in F2 populations.

		Observed		Expected			
Population	NO. plants	WT	*bzu2-1*	WT	*bzu2-1*	*x*^2^	P-values
F2	3296	2467	829	2472	824	0.0404^a^	0.9<P<0.95

*x*^*2*^_0.95,1_ = 0.0039; *x*^*2*^_0.50,1_ = 0.15

^a^There was no significant difference between the observed and expected number of plants.

### Map-based cloning for *BZU2*

The homozygous *bzu2-1* (-/-) mutant is lethal. We use the heterozygous *bzu2-1* (+/-) crossed with B73 for generation of the reciprocal F1 population. The F2 population resulting from the self-crossed F1, and a map-based cloning population, was screened for the *bzu2-1* phenotype from the F2 population (S2 Fig). Preliminary mapping of *BZU2* used 306 plants from the F2 population, derived from a cross between Mo17 and B73. The 384 SSR markers (SIGMA catalog number M4193) selected from the Maize Genetics and Genomic Database cover the entire genome with an average of 20 cM units of map distance between every two SSR markers. More SSR markers that were genetically mapped on IBM2 2008 Neighbors Frame were used, and *BZU2* was mapped between SSR markers bnlg1863 (recombination rate 2.1%) and umc1858 (recombination rate 12.5%). Based on Maize B73_RefGen_v3 (http://www.maizesequence.org), the *BZU2* was mapped to the chromosome VIII between bins 8.03 and 8.04. Therefore, all BAC contigs in bins 8.03–8.04 were exploited to develop new polymorphic markers. To develop more SSR markers, SSR Hunter 1.3 [45] was used to search for SSR sequences present in bins 8.03–8.04. SSRs and their flanking sequences about 150 bp were then aligned with NCBI nucleotide BLAST (http://www.ncbi.nlm.nih.gov) (high-throughput genomic sequences: HTGSs). Only single sequences were used as SSR markers and amplified by PCR. In fine mapping, ~7,000 plants from F2 population deprived from a cross between Mo17 and B73, 63 polymorphic markers were used, *BZU2* was mapped in SSR markers 79M15 (79.01 M) and 79M45 (79.70 M). Further analysis and sequencing confirm that GRMZM2G417164 was located between these two markers, AGCT insert in the + 390 bp of GRMZM2G417164.

### Generation of CRISPR Lines

CRISPR constructs were designed using the vector system and following the design protocol [46], and was done by Genovo Biotechnology Co (Xi’an, Shanxi). The PAM sites were chosen at the + 289 bp downstream of the start codon of *BZU2/ZmMUTE* genome region, because there is no intron in *BZU2/ZmMUTE*. The sequence of the gRNA is CCTGTCATGATCAAGGAGCTCGC (S1 Table). To genotype CRISPR-induced mutations, we amplified a 668-bp fragment including the guide RNA site by PCR from the genome of transgenic seedlings [47, 48], and the PCR products were sequenced. We obtained three CRISPR/Cas9 mutant lines: *bzu2-2*, *bzu2-3* and *bzu2-4*. A 5 bp and 25 bp deletion was detected behind PAM site in *bzu2-2* line and *bzu2-3* respectively, while a 1 bp insertion was detected behind PAM site *bzu2-4* line. In T0 mutant plants, the phenotype of homozygous mutants lines was comparable to *bzu2-1* (Fig 4C). The heterozygous T0 mutants lines were planted in the field to get seeds. In the T1 populations, homozygous mutants lines also showed a similar phenotype to that of *bzu2-1*.

### Reporter constructs

Reporter constructs to be transformed into *Oryza sativa Japonica* were generated using the In-fusion cloning with the monocot binary expression vector pIPKb003 and to be transformed in *Arabidopsis thaliana* were generated using the Gateway Recombination Cloning Technology with plant expression vector pGWB504. *AtMUTE* promoter and genome sequences were amplified from the *Arabidopsis* (Col-0) genome, *OsMUTE* promoter and genome sequences were amplified from the *Japonica* genome, and *BdMUTE* sequence was amplified from the *Brachypodium distachyon* genome. All genomic DNA samples were produced using the Plant Genomic DNA Kit (TIANGEN). RNA samples were produced using the TRIzol extraction method and corresponding cDNA was obtained by M-MLV reverse transcriptase (M1705, Promega).

The *Pri1* and *Pri2* primers were used to clone the *OsMUTE* promoter from the rice genome. We then amplified tag-YFP using forward primers *Pri3* and *Pri4* (for *AtMUTE*), *Pri3* and *Pri7* (for *OsMUTE*), *Pri3* and *Pri10* (for *BZU2/ZmMUTE*), and *Pri3* and *Pri14* (for *BdMUTE*), respectively, which have sequences homologous with the *OsMUTE* promoter, and using four reverse primers (*Pri4*, *Pri7*, *Pri10*, *Pri14*) to get four tag-YFP PCR products which has homologous sequence with the *AtMUTE*, *OsMUTE*, *BZU2/ZmMUTE* and *BdMUTE*. Then we used primers *Pri5* and *Pri6* to clone the *AtMUTE* ORF carrying a STOP codon, primers *Pri8* and *Pri9* to clone the *OsMUTE* ORF with a STOP codon, and the primers *Pri11* and *Pri12* to clone the *ZmMUTE* ORF with a STOP codon. Primers *Pri11* and *Pri13* were used to clone the *ZmMUTE* ORF with a deletion of 30 AA in the C terminal replaced by a STOP codon, and primers *Pri15* and *Pri16* to clone the *BdMUTE* ORF with a STOP codon. Primers *Pri15* and *Pri17* were used to clone the *BdMUTE* ORF with a deletion of 30 AA in the C terminal replaced by a STOP codon. The three PCR products were employed for in-fusion cloning using the vector *pIPKb003*. *OsMUTEp*:*YFP-AtMUTE*, *OsMUTEp*:*YFP-OsMUTE*, *OsMUTEp*:*YFP-BdMUTE* and *OsMUTEp*:*YFP-BZU2/ZmMUTE* were produced according to the procedures described in the manual of the Clone Express® MultiS One Step Cloning Kit (Vazyme Biotech Co., Ltd, China).

*Pri18* and *Pri19* were used to clone the whole genomic fragment of *AtMUTEp*:*AtMUTE* from the *Arabidopsis thaliana* (Col-0) genome. The tag-YFP fragment was amplified using *Pri20* and *Pri21*, *Pri20* bearing homology to *AtMUTE* and *Pri21* providing an *attB2* site. The fusion gene *AtMUTEp*:*AtMUTE-YFP* was then generated by overlap PCR using primers *Pri18* and *Pri21*, and then BP recombined into pDONR207, next LR recombined into destination vector pGWB504. For *AtMUTEp*:*YFP-BZU2/ZmMUTE* and *AtMUTEp*:*YFP-BdMUTE*, primers *Pri18* and *Pri22* were used to clone the *AtMUTE* promoter from the *Arabidopsis* (Col-0) genome. *Pri22* provided sequence homologous to tag-YFP. With primer *Pri23* and reverse primers (*Pri24*, *Pri25*), this amplified the tag-YFP PCR products all carrying sequences homologous to *BZU2/ZmMUTE* and *BdMUTE*. We then used primers *Pri26* and *Pri27* to clone the *BZU2/ZmMUTE* ORF carrying a STOP codon, and primers *Pri26* and *Pri28* to clone the *BZU2/ZmMUTE* ORF with 30 AA at the C terminal being replaced with a STOP codon. Primers *Pri29* and *Pri30* were similarly used to clone the *BdMUTE* ORF with a STOP codon, and primers *Pri29* and *Pri31* to clone of *BdMUTE* ORF with 30 AA at the C terminal being replaced with a STOP codon. All reverse primers amplified ORFs carrying Gateway *attB2* sites. The inframe gene fusions *AtMUTEp*:*YFP-BZU2/ZmMUTE*, *AtMUTEp*:*YFP-BZU2/ZmMUTE-ΔC*, *AtMUTEp*:*YFP-BdMUTE*, and *AtMUTEp*:*YFP-BdMUTE-ΔC* were generated by overlap PCR using the forward primer *Pri18* and four reverse primers (*Pri27*, *Pri28*, *Pri30*, *Pri31*), then BP recombined into pDONR207, followed by LR recombined into the destination vector pGWB504. *AtMUTEp*:*AtMUTE-YFP*, *AtMUTEp*:*YFP-BZU2/ZmMUTE*, *AtMUTEp*:*YFP-BdMUTE*, *AtMUTEp*:*YFP-BZU2/ZmMUTE-*ΔC and *AtMUTEp*:*YFP-BdMUTE-*ΔC were produced through Gateway Recombination Cloning (Invitrogen).

*BZU2/ZmMUTEp*:*YFP-BZU2/ZmMUTE* was inserted into the maize genome via *Agrobacterium* mediated transformation (see below). The promoter of *BZU2/ZmMUTE* being amplified from the wild-type (Mo17) genome using primers *Pri32* and *Pri33*, the YFP ORF lacking the stop codon amplified using primers *Pri34* and *Pri35*, and the ORF of *BZU2/ZmMUTE* including the stop codon amplified from the cDNA of Mo17 with primers *Pri36* and *Pri37*. The three PCR products were employed for In-fusion cloning with the pCM3300 vector. The primers used in this work are listed in S1 Table.

### Generation and analysis of transgenic lines

Reporter constructs of *OsMUTEp*:*YFP-AtMUTE*, *OsMUTEp*:*YFP-OsMUTE*, *OsMUTEp*:*YFP-BdMUTE*, *OsMUTEp*:*YFP-BZU2/ZmMUTE*, *OsMUTEp*:*YFP-BZU2/ZmMUTE*-ΔC and *OsMUTEp*:*YFP-BdMUTE*-ΔC were transformed into *Oryza sativa Japonica* calli with EHA105 *Agrobacterium*, as previously described [49]. *AtMUTEp*:*AtMUTE-YFP*, *AtMUTEp*:*YFP- BZU2/ZmMUTE*, *AtMUTEp*:*YFP-BdMUTE*, *AtMUTEp*:*YFP-BZU2/ZmMUTE-*ΔC and *AtMUTEp*:*YFP-BdMUTE-*ΔC were transformed into *Arabidopsis* (Col-0) using *Agrobacterium* strain GV3101 [50].

Maize transgenic lines were produced via *Agrobacterium*-mediated transformation [51]. Maize seedlings were grown in the greenhouse or in the experimental field. Ears containing immature embryos, between 1.0 to 2.0 mm in length along the axis and optimal for transformation, were collected 8 to 13 days after pollination. The immature embryos were submerged in an *Agrobacterium tumefaciens* suspension contained in a 2.0 mL Eppendorf tube at room temperature for 1 h. The solution was then removed, and the embryos transferred onto fresh co-cultivation solid medium with the scutellum face up, and were incubated in darkness at 25°C for 2–3 days. After that, the calli were transferred onto fresh screen solid medium, screening three times for a period of 2 weeks each. The Type I calli that further proliferated were transferred to shoot regeneration medium, and incubated under continuous illumination (5,000 lux) at 25°C for 14–30 days. The emerging shoots were transferred onto root regeneration medium, and were incubated under continuous illumination (5,000 lux) at 25°C for 14–30 days. The rooted seedlings were transferred to pots containing appropriately supplemented soil for growth in the greenhouse for 3–4 months to collect progeny seeds.

We typically analyzed at least three independent lines in the T0 generation (depending on how many independent lines were recovered upon regeneration) and confirmed the observed expression pattern in at least three T1 individuals if the transgenics were fertile and produced seeds. T1 maize transgenic plants were used in this study. The images were acquired using a Leica SP8 confocal microscope, the cellular membranes being counterstained with propidium iodide (PI, red) in maize and *Arabidopsis*, and FM4-64 (red) in rice.

### Analysis of stomatal complex development using microscopy

To determine the stomatal phenotype in wild-type and *bzu2-1* mutants (Fig 1D), epidermal strips were peeled from mature leaves of wild-type and *bzu2-1* plants, and were observed using a Zeiss Axioskop II microscope equipped with differential interference contrast optics. To obtain images characterizing the process of stomatal complex development (Fig 2A), around ~1.5 cm of segments from the leaf base were excised from 8-day-old seedlings. The tissues were cleared in Herr's solution (lactic acid:chloral hydrate:phenol:clove oil:xylene (2:2:2:2:1, by weight)) [52]. The stomatal development process was studied using Zeiss Axioskop II microscope equipped with differential interference contrast optics.

For F-actin observation, ~1.5 cm (the section of stomatal complex develops from stomatal lineage cell to mature stomatal complex is about 1.5 cm base in the leaf from maize seedling root node, indicated by the analysis of stomatal complex development using microscopy) of basal leaf segments, excised from 8-day-old seedlings, were cut into 0.2 cm wide x 0.5 cm long strips, and fixed for 30 min at room temperature in a solution comprising 4% paraformaldehyde, dissolved in 50 mM PEM (50 mM PIPES, 2.5 mM EGTA, 2.5 mM MgCl_2_). The strips were washed three times for 5 min in 50 mM PEM, and were permeabilized by submersion 20 mins in 50 mM PEM containing 5% DMSO and 1% Triton X-100. After three further washes in 50 mM PEM, the sections were incubated for 1.5 h in 50 mM PEM solution containing 90 nM AlexaFluor 488-phalloidin (dissolved in DMSO, Invitrogen/Molecular Probes) at room temperature. Images were acquired using a Zeiss LSM710 confocal microscope.

For observation of the polarized nuclei of the GMCs, ~1.5 cm of second or third basal leaf segments excised from 8-day-old seedlings was cut into 0.2 cm wide x 0.5 cm long strips directly stained with 4 μg/mL Hoechst 33258 (94403-1ML, SIGMA) dissolved in water for 15 min at room temperature. Images were acquired using a Leica SP8 Confocal Microscope.

### BZU2/ZmMUTE antibody production

A peptide corresponding to amino acids 192–206 of the BZU2/ZmMUTE protein (GQDTAEQKPQAEENH) was synthesized, conjugated to KLH, and used for polyclonal antibody production in rabbits by the Hanlin Biotechnology Co. (Shijiazhuang, Hebei).

### Chromatin immunoprecipitation

ChIP-qPCR experiments were carried out using the Magna ChIP kit (MAGNA001, Millipore) with minor modification [53, 54]. Samples (0.4 g) of the stomatal development zones of 8-day-old seedlings were collected, and then immersed in buffer A (0.4 M sucrose, 10 mM Tris [pH 8], 1 mM EDTA, 1 mM PMSF) containing 1% formaldehyde, and were subjected to four eight-minute cycles under vacuum, until the materials became translucent. The materials were then transferred to fresh buffer A containing 0.1 M glycine, and incubation was continued for 16 min at 4°C. The materials were then washed, and frozen in liquid nitrogen. Samples (approximately 0.4 g) of the materials were ground for each immunoprecipitation, and were resuspended in 1 mL lysis buffer (50 mM HEPES [pH 7.5], 150 mM NaCl, 1 mM EDTA, 1% Triton X-100, 0.1% deoxycholate, 0.1% SDS, 1 mM PMSF, and 1 x Roche Protease Inhibitor Cocktail), followed by immunoprecipitation with an anti-GFP antibody (ab290, Abcam). Immunoprecipitated products were resuspended in 50 μL elution buffer. Approximately 3–5 μL was used for ChIP-qPCR. Each immunoprecipitation was performed three times independently, with the wild-type being used as the control. The primers for ChIP-qPCR are listed in S1 Table.

### Yeast one-hybrid assay

The plasmids pB42AD-BZU2/ZmMUTE and pB42AD-BZU2/ZmMUTE-ΔC, and wild-type and mutated PAN1-P1:LacZi, PAN2-P2:LacZi, PAN2-P3:LacZi were co-transformed into yeast strain EGY48 using standard transformation techniques. Transformants were grown on dropout plates containing X-gal (5-Bromo-4-chloro-3-indolyl-β-D-galactopyranoside) for blue color development. pB42AD-SPL9 (squamosa promoter binding protein-like 9) reacting with DFR (dihydroflavonol reductase) [55] was used as a positive control, and combinations with the empty pB42AD vector were used as negative controls. The *PAN1* and *PAN2* primers are listed in S1 Table.

### EMSA

The EMSA was conducted using the LightShift™ Chemiluminescent EMSA Kit (Thermo Fisher Scientific) according to the manufacturer’s protocol. The coding sequence of *BZU2/ZmMUTE* was cloned into pGEX-2TK. Recombinant BZU2/ZmMUTE protein was expressed and purified from BL21 *E*. *coli*. The probes of the *PAN1/2* promoter were obtained by gene synthesis and biotin-labeled at their 5’ terminal. Biotin-unlabeled probes of the same sequences were used as competitors. The probe sequence is described in S1 Table.

### Sequences alignment and phylogenetic analysis

The amino acid sequences of BZU2/ZmMUTE and other homologous proteins were retrieved from NCBI (https://www.ncbi.nlm.nih.gov/). Alignments of BZU2/ZmMUTE (GRMZM2G417164) (in wild-type and *bzu2-1*), BdMUTE (LOC100843821), OsMUTE (Os05g51820) and AtMUTE (At3g06120) were conducted using the MUSCLE and BOXSHADE programs. Construction of phylogenic trees using the neighbor-joining method and confirmation of tree topology by bootstrap analysis (5,000 replicates) was performed with MEGA6 software (using default settings except for the replicates of bootstrap value) [56].

### RNA extraction and RT-qPCR

Samples were taken from ~1.5 cm of the base of leaves of 8-day-old maize seedlings for RT-qPCR analysis. Total RNA was isolated using the TRIzol reagent (Life Technologies) according to the manufacturer’s protocol. Reverse transcription into cDNA was done with 2 μg total RNA in 25 μL reverse transcription mixture, using M-MLV Reverse Transcriptase (M1705, Promega). The cDNA was diluted to 100 μL, and 1 μL diluted cDNA was used as the template for quantitative RT-PCR analysis. The maize *ZmUbiquitin 2* gene was used as an internal standard to normalize expression of the tested genes. Quantitation was performed using at least three independent biological replicates [57]. The primers used for RT-qPCR are listed in S1 Table.

### Subcellular localization

The coding sequences of *BZU2/ZmMUTE* was cloned into pGreen0280 (*35S*:*BZU2/ZmMUTE-GFP*) for the analysis of subcellular localization. *35S*:*BZU2/ZmMUTE-GFP*, empty vector, and *35S*:*H2B-mCherry* were cotransfected into tobacco leaves. Green and red fluorescence was imaged using the Zeiss LSM710 confocal microscope 24 h after *Agrobacterium*-mediated infiltration of tobacco (*Nicotiana benthamiana*) leaves [58]. The primers used are listed in S1 Table.

### E-box *cis*-element analysis

Previous studies indicated that the E-box *cis*-element is the conserved CANNTG. The *cis*-element was produced by the Multiple Expectation Maximization for Motif Elicitation MEME Suite web server [36].

## Supporting information

S1 Fig Image and quantitative analysis of subsidiary cells in wild-type and *bzu2-1* plants. (A) Representative images illustrating the first leaf epidermis of wild-type and *bzu1-2* seedlings. Scale bars, 50 μm. The arrow indicates the abnormal subsidiary cell. (B) In the first leaves of *bzu2-1* plants, 95.39% of the stomatal complexes have no subsidiary cells, and only 4.61% of the stomatal complexes have one subsidiary cell. Error bars indicate SD, n = 802, **P<0.01, Student’s *t* test.
(TIF)

S2 Fig Construction of the map-based cloning population. The homozygous *bzu2-1* (-/-) mutant is lethal. We therefore used the heterozygote *bzu2-1* (+/-) crossed to B73 for generation of reciprocal F1 hybrid progeny. F2 individuals resulting from the self-crossed F1 and from the map-based cloning population were screened for the *bzu2-1* mutant phenotype within the F2 population.
(TIF)

S3 Fig Phylogenetic analysis of the BZU2/ZmMUTE proteins from different species. The numbers above or below the branches are the bootstrap values from 5,000 replicates. BZU2/ZmMUTE, AtMUTE and BdMUTE are boxed. NCBI reference sequence numbers are indicated behind the gene names. The species of origin of the MUTE are indicated by the abbreviation preceding the gene names: Ac, *Ananas comosus*; At, *Aegilops tauschii*; At, *Arabidopsis thaliana*; Bd, *Brachypodium distachyon*; Bn, *Brassica napus*; Ca, *Capsicum annuum*; Cs, *Cucumis sativus*; Dc, *Dendrobium catenatum*; Eg, *Elaeis guineensis*; Gh, *Gossypium hirsutum*; Gm, *Glycine max*; Gr, *Gossypium raimondii*; In, *Ipomoea nil*; Jr, *Juglans regia*; Md, *Malus domestica*; Nn, *Nelumbo nucifera*; Nt, *Nicotiana tomentosiformis*; Os, *Oryza sativa*; Pe, *Populus euphratica*; Pg, *Punica granatum*; Pp, *Prunus persica*; Pt, *Populus trichocarpa*; Sb, *Sorghum bicolor*; Si, *Sesamum indicum*; Si, *Setaria italica*; Sl, *Solanum lycopersicum*; Sm, *Salvia miltiorrhiza*; St, *Solanum tuberosum*; St, *Solanum tuberosum*; Va, *Vigna angularis*; Vv, *Vitis vinifera*; Zm, *Zea mays*.
(TIF)

S4 Fig *bzu2* mutants generated by CRISPR/Cas9 system. (A) Diagram representing the gRNA target used in the CRISPR/Cas9 system to generate *bzu2* mutants. The PAM sequence is located at position +289 nt in *BZU2*. (B-D) Sequencing confirmation of *bzu2* mutants. The mutations in *bzu2-2* (B), *bzu2-3* (C) and *bzu2-4* (D) in the T0 generation.
(TIF)

S5 Fig Subcellular localization of BZU2/ZmMUTE in tobacco epidermal cells. *35S*:*GFP* and *35S*:*BZU2/ZmMUTE-GFP* were transiently expressed in tobacco leaves. Green and red fluorescence was imaged using confocal microscopy 24 h after *Agrobacterium*-mediated infiltration. *35S*:*H2B-mCherry* serves as a nuclear marker. Scale bars, 50 μm.
(TIF)

S6 Fig Confocal images of *OsMUTEp*:*YFP-MUTE* expression at different stages of stomatal development in rice seedlings. Confocal images of *OsMUTEp*:*NLS-YFP* and *OsMUTEp*:*YFP-MUTE* expression (yellow) with FM4-64 counterstaining to visualize the outlines of the plasma membranes (red) at the bases of the second and third leaves. White arrows in (A) to (D) indicate the SMC and SC. (A) Expressions of *OsMUTEp*:*NLS-YFP* which is only located in the nuclei of GMCs; after the formation of SCs, the signal disappeared from mature guard cells and subsidiary cells. (B) *OsMUTEp*:*YFP-OsMUTE* expression at the different stages of stomatal development. (C) *OsMUTEp*:*YFP-ZmMUTE* expression at the different stages of stomatal development. *OsMUTEp*:*YFP-ZmMUTE* starts to be expressed in the early GMCs, reaching its peak in GMCs, but also being expressed in SMCs. The YFP signal is maintained until after GMC division, finally disappearing during stomatal maturation. (D) *OsMUTEp*:*YFP-BdMUTE* expression in different stages of stomatal development. (E) *OsMUTEp*:*YFP-AtMUTE* expression at different stages of stomatal development. The fluorescence of *YFP-AtMUTE* was very weak in the early GMCs, and was not detected in SMCs. Confocal images of *OsMUTEp*:*YFP-ZmMUTE*-ΔC (F) and *OsMUTEp*:*YFP- BdMUTE*-ΔC (G) show that *YFP-ZmMUTE*-ΔC and *YFP-BdMUTE*-ΔC are only found in the early GMCs, and not in the SMCs and young SCs at the base of the second and third leaves. All images are from second or third leaf segments of the T0 (B, C, D and E) and T1 (A, F and G) generations of transgenic rice. Scale bars, 10 μm.
(TIF)

S7 Fig Yeast one-hybrid assays showing BZU2/ZmMUTE-ΔC binding to the E-box motifs present in the *PAN1* and *PAN2* promoters. (TIF)

S8 Fig EMSA assays showing specific binding of BZU2/ZmMUTE-ΔC to the E-box motifs in the *PAN1* and *PAN2* promoters. EMSA was performed with probes that were biotin-labeled (biotin probe) or that were unlabeled (cold probe), E-box-containing DNA fragments (top panel) and recombinant BZU2/ZmMUTE-ΔC protein; specific combinations are shown above the autoradiograph. Unlabeled fragments were added gradually in 2-, 5- or 10- fold excess as indicated.
(TIF)

S9 Fig Comparative RT-qPCR analysis of the expression of *PAN1*, *PAN2* and *ZmFAMA* in leaf bases. RNA was extracted from leaf segments representing the region up to ~1.5 cm from the leaf bases of the second and third leaves (taken 4 days after germination, and at the point of second leaf emergence). Transcript level is decreased in the *bzu2-1* mutant as compared to the wild-type. RT-qPCR values are expressed as the mean ± SD compared to that of the internal control (*ZmUbiquitin 2*). Error bars indicate SD, n = 3, Student’s *t* test, **P<0.01. Assays were done with triplicate repeats.
(TIF)

S10 Fig Western blot of *BZU2/ZmMUTEp:YFP-BZU2/ZmMUTE* transgenic plants and wild-type using anti-GFP antibody. 20 μg of total protein taken from the second and third leaf bases of 8-day-old seedlings of *BZU2/ZmMUTEp*:*YFP-BZU2/ZmMUTE* transgenic plants and wild-type, was separated by electrophoresis. The corresponding blot was incubated in primary antibody (anti-GFP, ab290, Abcam) at a dilution 1:10,000, actin being used as a control.
(TIF)

S11 Fig Western blot analysis of BZU2/ZmMUTE antibody and ChIP-qPCR analysis of BZU2/ZmMUTE binding to the promoters of *PAN1* and *PAN2*. (A) 20 μg of total protein taken from the second and third leaf bases of 8-day-old wild-type and *bzu2-1* mutant seedlings. Western blot results indicate that BZU2/ZmMUTE cannot be detected in the *bzu2-1* mutant. (B) ChIP-qPCR results showing that the promoter fragments of *PAN1* and *PAN2* can be amplified from the immunoprecipitation pulled down by the anti-BZU2/ZmMUTE antibody. The sequences used for ChIP-qPCR contain E-box *cis*-elements from within the *PAN1* and *PAN2* promoter regions. Samples were harvested for the chromatin immunoprecipitation (ChIP) experiment taken from a region extending ~ 1.5 cm from the base of the second and third leaves of 8-day-old seedlings with (+Ab) or without (-Ab) addition of an anti-BZU2/ZmMUTE antibody. *Zm178* (GRMZM2G134178), a gene not involved in stomatal development, was used as a negative control. Error bars indicate SD, n = 3, Student’s *t* test, **P<0.01.
(TIF)

S1 Table Primers used in this study. (XLSX)

## References

[1] Jezek M, Blatt MR. The membrane transport system of the guard cell and its integration for stomatal dynamics. Plant Physiol. 2017;174(2):487–519. 10.1104/pp.16.01949 28408539 PMC5462021

[2] Chen ZH, Chen G, Dai F, Wang Y, Hills A, Ruan YL, et al Molecular evolution of grass stomata. Trends Plant Sci. 2017;22(2):124–139. 10.1016/j.tplants.2016.09.005 27776931

[3] Vaten A, Bergmann DC. Mechanisms of stomatal development: an evolutionary view. Evodevo. 2012;3(1):11 10.1186/2041-9139-3-11 22691547 PMC3390899

[4] Galatis B, Apostolakos P. The role of the cytoskeleton in the morphogenesis and function of stomatal complexes. New Phytol. 2004;161 (3): pp. 613–639. 10.1046/j.1469-8137.2003.00986.x 33873710

[5] Farquharson KL. Polarization of subsidiary cell division in maize stomatal complexes. Plant Cell. 2012;24(11): pp. 4313.

[6] Stebbins GL, Shah SS. Developmental studies of cell differentiation in the epidermis of monocotyledons. Dev Biol. 1960;2(6): pp. 477–500. 10.1016/0012-1606(62)90036-2 13862290

[7] Han SK, Torii KU. Lineage-specific stem cells, signals and asymmetries during stomatal development. Development. 2016;143(8):1259–1270. 10.1242/dev.127712 27095491

[8] Qi X, Han SK, Dang JH, Garrick JM, Ito M, Hofstetter AK, et al Autocrine regulation of stomatal differentiation potential by EPF1 and ERECTA-LIKE1 ligand-receptor signaling. Elife. 2017;6 10.7554/eLife.24102 28266915 PMC5358980

[9] Pillitteri LJ, Torii KU. Breaking the silence: three bHLH proteins direct cell-fate decisions during stomatal development. Bioessays. 2007;29(9):861–870. 10.1002/bies.20625 17691100

[10] MacAlister CA, Ohashi-Ito K, Bergmann DC. Transcription factor control of asymmetric cell divisions that establish the stomatal lineage. Nature. 2007;445(7127):537–540. 10.1038/nature05491 17183265

[11] Ohashi-Ito K, Bergmann DC. *Arabidopsis* FAMA controls the final proliferation/differentiation switch during stomatal development. Plant Cell. 2006;18(10):2493–2505. 10.1105/tpc.106.046136 17088607 PMC1626605

[12] Pillitteri LJ, Sloan DB, Bogenschutz NL, Torii KU. Termination of asymmetric cell division and differentiation of stomata. Nature. 2007;445(7127):501–505. 10.1038/nature05467 17183267

[13] Wu Z, Chen L, Yu Q, Zhou W, Gou X, Li J, et al Multiple transcriptional factors control stomata development in rice. New Phytol. 2019;223(1):220–232. 10.1111/nph.15766 30825332

[14] Robinson S, Barbier de Reuille P, Chan J, Bergmann D, Prusinkiewicz P, Coen E. Generation of spatial patterns through cell polarity switching. Science. 2011;333(6048):1436–1440. 10.1126/science.1202185 21903812 PMC3383840

[15] Han SK, Qi X, Sugihara K, Dang JH, Endo TA, Miller KL, et al MUTE directly orchestrates cell-state switch and the single symmetric division to create stomata. Dev Cell. 2018;45(3):303–315 e5. Epub 2018/05/09. 10.1016/j.devcel.2018.04.010 29738710

[16] Pillitteri LJ, Bogenschutz NL, Torii KU. The bHLH protein, MUTE, controls differentiation of stomata and the hydathode pore in *Arabidopsis*. Plant Cell Physiol. 2008;49(6):934–943. 10.1093/pcp/pcn067 18450784

[17] Bhave NS, Veley KM, Nadeau JA, Lucas JR, Bhave SL, Sack FD. TOO MANY MOUTHS promotes cell fate progression in stomatal development of *Arabidopsis* stems. Planta. 2009;229(2):357–367. 10.1007/s00425-008-0835-9 18979118

[18] Zhang L, Hu G, Cheng Y, Huang J. Heterotrimeric G protein alpha and beta subunits antagonistically modulate stomatal density in *Arabidopsis thaliana*. Dev Biol. 2008;324(1):68–75. 10.1016/j.ydbio.2008.09.008 18834874

[19] Raissig MT, Abrash E, Bettadapur A, Vogel JP, Bergmann DC. Grasses use an alternatively wired bHLH transcription factor network to establish stomatal identity. Proc Natl Acad Sci USA. 2016;113(29):8326–8331. 10.1073/pnas.1606728113 27382177 PMC4961163

[20] Peterson KM, Rychel AL, Torii KU. Out of the mouths of plants: the molecular basis of the evolution and diversity of stomatal development. Plant Cell. 2010;22(2):296–306. 10.1105/tpc.109.072777 20179138 PMC2845417

[21] Raissig MT, Matos JL, Gil MX, Kornfeld A, Bettadapur A, Abrash E, et al Mobile MUTE specifies subsidiary cells to build physiologically improved grass stomata. Science. 2017;355(6330):1215–1218. 10.1126/science.aal3254 28302860

[22] Cartwright HN, Humphries JA, Smith LG. PAN1: a receptor-like protein that promotes polarization of an asymmetric cell division in maize. Science. 2009;323(5914):649–651. 10.1126/science.1161686 19179535

[23] Gallagher K, Smith LG. Roles for polarity and nuclear determinants in specifying daughter cell fates after an asymmetric cell division in the maize leaf. Curr Biol. 2000;10(19):1229–1232. 10.1016/s0960-9822(00)00730-2 11050395

[24] Zhang X, Facette M, Humphries JA, Shen Z, Park Y, Sutimantanapi D, et al Identification of PAN2 by quantitative proteomics as a leucine-rich repeat-receptor-like kinase acting upstream of PAN1 to polarize cell division in maize. Plant Cell. 2012;24(11):4577–4589. 10.1105/tpc.112.104125 23175742 PMC3531853

[25] Humphries JA, Vejlupkova Z, Luo A, Meeley RB, Sylvester AW, Fowler JE, et al ROP GTPases act with the receptor-like protein PAN1 to polarize asymmetric cell division in maize. Plant Cell. 2011;23(6):2273–2284. 10.1105/tpc.111.085597 21653193 PMC3160025

[26] Facette MR, Park Y, Sutimantanapi D, Luo A, Cartwright HN, Yang B, et al The SCAR/WAVE complex polarizes PAN receptors and promotes division asymmetry in maize. Nat Plants. 2015;1:14024 10.1038/nplants.2014.24 27246760

[27] Sutimantanapi D, Pater D, Smith LG. Divergent roles for maize PAN1 and PAN2 receptor-like proteins in cytokinesis and cell morphogenesis. Plant Physiol. 2014;164(4):1905–1917. 10.1104/pp.113.232660 24578508 PMC3982752

[28] Gao Z, Liu H, Wang H, Li N, Wang D, Song Y, et al Generation of the genetic mutant population for the screening and characterization of the mutants in response to drought in maize. Chinese Sci Bull. 2014;59(8):766–775. 10.1007/s11434-013-0031-6 PMID: 243442

[29] Merlot S, Mustilli AC, Genty B, North H, Lefebvre V, Sotta B, et al Use of infrared thermal imaging to isolate *Arabidopsis* mutants defective in stomatal regulation. Plant J. 2002;30(5):601–609. 10.1046/j.1365-313X.2002.01322.x 12047634

[30] Lewis MW, Bolduc N, Hake K, Htike Y, Hay A, Candela H, et al Gene regulatory interactions at lateral organ boundaries in maize. Development. 2014;141(23):4590–4597. 10.1242/dev.111955 25359728

[31] Frank MJ, Cartwright HN, Smith LG. Three Brick genes have distinct functions in a common pathway promoting polarized cell division and cell morphogenesis in the maize leaf epidermis. Development. 2003;130(4):753–762. 10.1242/dev.00290 12506005

[32] Frank MJ, Smith LG. A small, novel protein highly conserved in plants and animals promotes the polarized growth and division of maize leaf epidermal cells. Curr Biol. 2002;12(10):849–853. 10.1016/s0960-9822(02)00819-9 12015123

[33] Kamiya N, Itoh J-I, Morikami A, Nagato Y, Matsuoka M. The SCARECROW gene's role in asymmetric cell division in rice plants. Plant J. 2003 36(1): pp. 45–54. 10.1046/i.1365-313x.2003.01856.x 12974810

[34] Liu T, Ohashi-Ito K, Bergmann DC. Orthologs of *Arabidopsis thaliana* stomatal bHLH genes and regulation of stomatal development in grasses. Development. 2009;136(13):2265–2276. 10.1242/dev.032938 19502487

[35] Lau OS, Davies KA, Chang J, Adrian J, Rowe MH, Ballenger CE, et al Direct roles of SPEECHLESS in the specification of stomatal self-renewing cells. Science. 2014;345(6204):1605–1609. 10.1126/science.1256888 25190717 PMC4390554

[36] Machanick P, Bailey TL. MEME-ChIP: motif analysis of large DNA datasets. Bioinformatics. 2011;27(12):1696–1697. 10.1093/bioinformatics/btr189 21486936 PMC3106185

[37] Davies KA, Bergmann DC. Functional specialization of stomatal bHLHs through modification of DNA-binding and phosphoregulation potential. Proc Natl Acad Sci USA. 2014;111(43):15585–15590. 10.1073/pnas.1411766111 25304637 PMC4217405

[38] Mineyuki Y, Palevitz BA. Relationship between preprophase band organization, F-actin and the division site in *Allium*. J Cell Sci. 1990;97(2): pp. 283.

[39] Cho S-O, Wick SM. Distribution and function of actin in the developing stomatal complex of *winter rye* (*Secale cereale cv*. *Puma*). Protoplasma. 1990;157(1): pp. 154–164.

[40] Palevitz BA. Comparative effects of phalloidin and cytochalasin B on motility and morphogenesis in *Allium*. Can J Bot. 1980;58(7): pp. 773–785.

[41] Palevitz BA, Hepler PK. The control of the plane of division during stomatal differentiation in *Allium*. Chromosoma. 1974;46(3): pp. 297–326.

[42] Kennard JL, Cleary AL. Pre-mitotic nuclear migration in subsidiary mother cells of Tradescantia occurs in G1 of the cell cycle and requires F-actin. Cell Motil Cytoskel. 1997;36(1):55–67. 10.1002/(SICI)1097-0169(1997)36:1<55::AID-CM5>3.0.CO;2-G 8986377

[43] Cleary AL, Mathesius U. Rearrangements of F-actin during stomatogenesis visualised by confocal microscopy in fixed and permeabilised *Tradescantia* leaf epidermis. Bot Act. 1996;109(1): pp. 15–24.

[44] Cleary AL. F-actin redistributions at the division site in living *Tradescantia* stomatal complexes as revealed by microinjection of rhodamine-phalloidin. Protoplasma. 1995;185(3): pp. 152–165.

[45] Li Q, Wan JM. SSRHunter: development of a local searching software for SSR sites. Yi Chuan. 2005;27(5):808–810. Epub 2005/11/01. 16257914

[46] Liang Z, Zhang K, Chen K, Gao C. Targeted mutagenesis in *Zea mays* using TALENs and the CRISPR/Cas system. J Genet Genomics. 2014;41(2):63–68. 10.1016/j.jgg.2013.12.001 24576457

[47] Liu H, Guo S, Lu M, Zhang Y, Li J, Wang W, et al Biosynthesis of DHGA12 and its roles in *Arabidopsis* seedling establishment. Nat Commun. 2019;10(1): pp. 1768 10.1038/s41467-019-09467-5 30992454 PMC6467921

[48] Guo J, Li K, Jin L, Xu R, Miao K, Yang F, et al A simple and cost-effective method for screening of CRISPR/Cas9-induced homozygous/biallelic mutants. Plant Methods. 2018;14:40 10.1186/s13007-018-0305-8 29872452 PMC5972395

[49] Hiei Y, Ohta S, Komari T, Kumashiro T. Efficient transformation of rice (*Oryza sativa* L.) mediated by *Agrobacterium* and sequence analysis of the boundaries of the T-DNA. Plant J. 1994;6(2):271–282. 7920717 10.1046/j.1365-313x.1994.6020271.x

[50] Clough SJ, Bent AF. Floral dip: a simplified method for *Agrobacterium*-mediated transformation of *Arabidopsis thaliana*. Plant J. 1998;16(6):735–743. . 10069079 10.1046/j.1365-313x.1998.00343.x

[51] Frame B, Main M, Schick R, Wang K. Genetic transformation using maize immature zygotic embryos. Methods Mol Biol. 2011;710:327–341. 10.1007/978-1-61737-988-8_22 21207278

[52] Herr JM Jr. A new clearing-squash technique for the study of ovule development in *Angiosperms*. Am J Bot. 1971;58(8): pp. 785–790.

[53] Wang YZ, Gu XF, Yuan WY, Schmitz RJ, He YH. Photoperiodic control of the floral transition through a distinct polycomb repressive complex. Dev Cell. 2014;28(6): pp. 727–736. 10.1016/j.devcel.2014.01.029 24613395

[54] Johnson LM, Cao XF, Jacobsen SE. Interplay between two epigenetic marks: DNA methylation and histone H3 lysine 9 methylation. Curr Biol. 2002;12(16): pp. 1360–1367. 10.1016/s0960-9822(02)00976-4 12194816

[55] Gou JY, Felippes FF, Liu CJ, Weigel D, Wang JW. Negative regulation of anthocyanin biosynthesis in *Arabidopsis* by a miR156-targeted SPL transcription factor. Plant Cell. 2011;23(4):1512–1522. 10.1105/tpc.111.084525 21487097 PMC3101539

[56] Tamura K, Stecher G, Peterson D, Filipski A, Kumar S. MEGA6: molecular evolutionary genetics analysis version 6.0. Mol Biol Evol. 2013;30(12):2725–2729. 10.1093/molbev/mst197 24132122 PMC3840312

[57] Guo S, Dai S, Singh PK, Wang H, Wang Y, Tan JLH, et al A membrane-bound NAC-like transcription factor OsNTL5 represses the flowering in *Oryza sativa*. Front Plant Sci. 2018;9:555 10.3389/fpls.2018.00555 29774039 PMC5943572

[58] Li K, Yang F, Zhang G, Song S, Li Y, Ren D, et al AIK1, A mitogen-activated protein kinase, modulates Abscisic Acid responses through the MKK5-MPK6 kinase cascade. Plant Physiol. 2017;173(2):1391–1408. 10.1104/pp.16.01386 27913741 PMC5291029

