# Supplementary figures and images for: BZU2/ZmMUTE controls symmetrical division of guard mother cell and specifies neighbor cell fate in maize
Source: PLoS Genet. 2019 Aug 29;15(8):e1008377. doi: 10.1371/journal.pgen.1008377 (PMC6738654; doi:10.1371/journal.pgen.1008377)

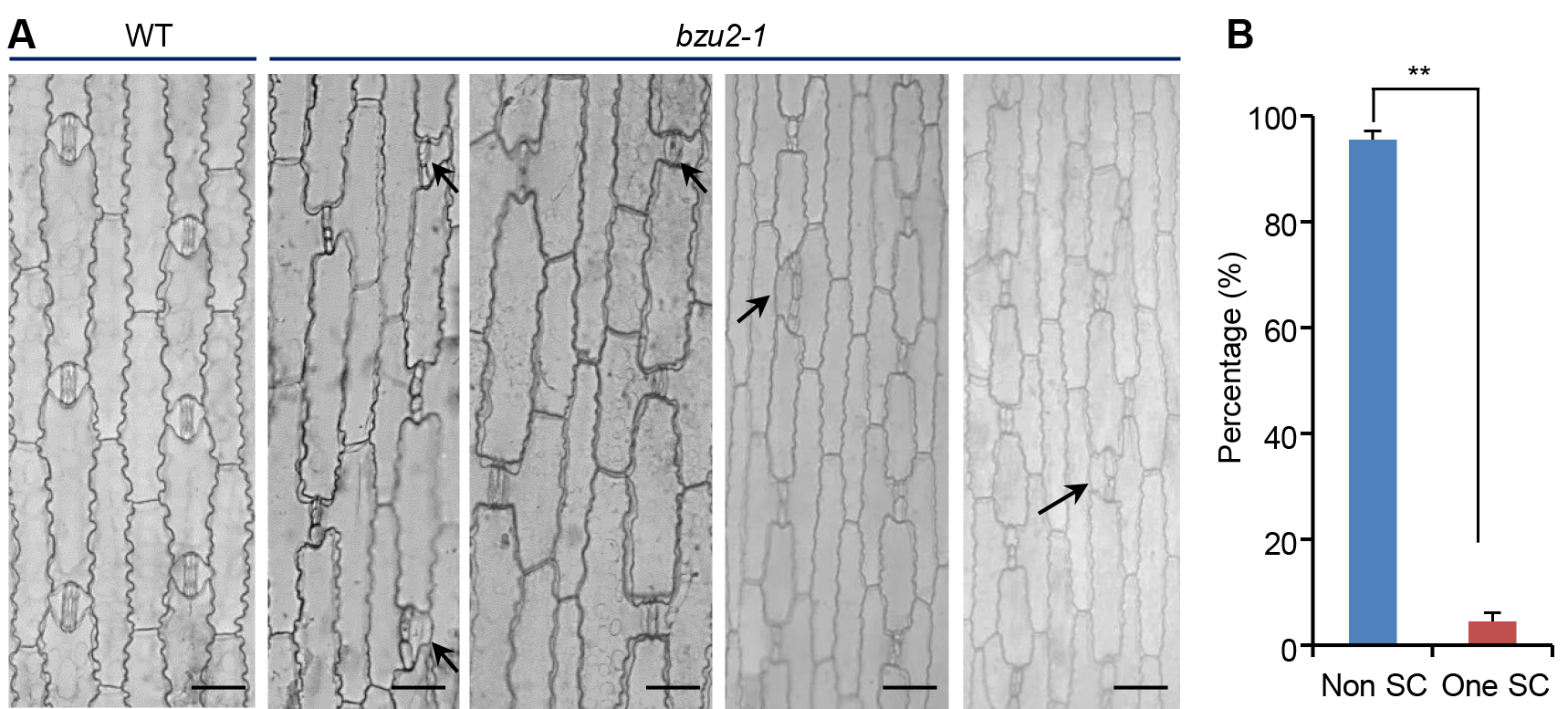

Supplement: S1 Fig — (A) Representative images illustrating the first leaf epidermis of wild-type and bzu1-2 seedlings. Scale bars, 50 μm. The arrow indicates the abnormal subsidiary cell. (B) In the first leaves of bzu2-1 plants, 95.39% of the stomatal complexes have no subsidiary cells, and only 4.61% of the stomatal complexes have one subsidiary cell. Error bars indicate SD, n = 802, **P<0.01, Student’s t test. (TIF) [file pgen.1008377.s001.tif]

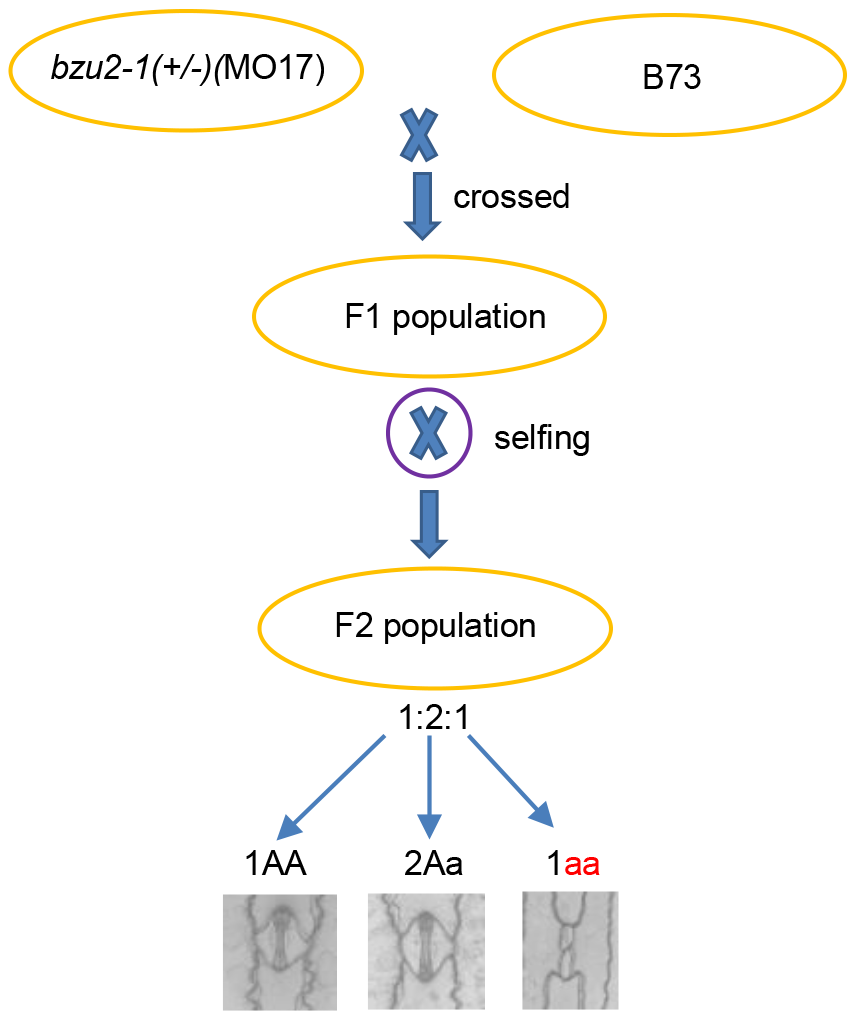

Supplement: S2 Fig — The homozygous bzu2-1 (-/-) mutant is lethal. We therefore used the heterozygote bzu2-1 (+/-) crossed to B73 for generation of reciprocal F1 hybrid progeny. F2 individuals resulting from the self-crossed F1 and from the map-based cloning population were screened for the bzu2-1 mutant phenotype within the F2 population. (TIF) [file pgen.1008377.s002.tif]

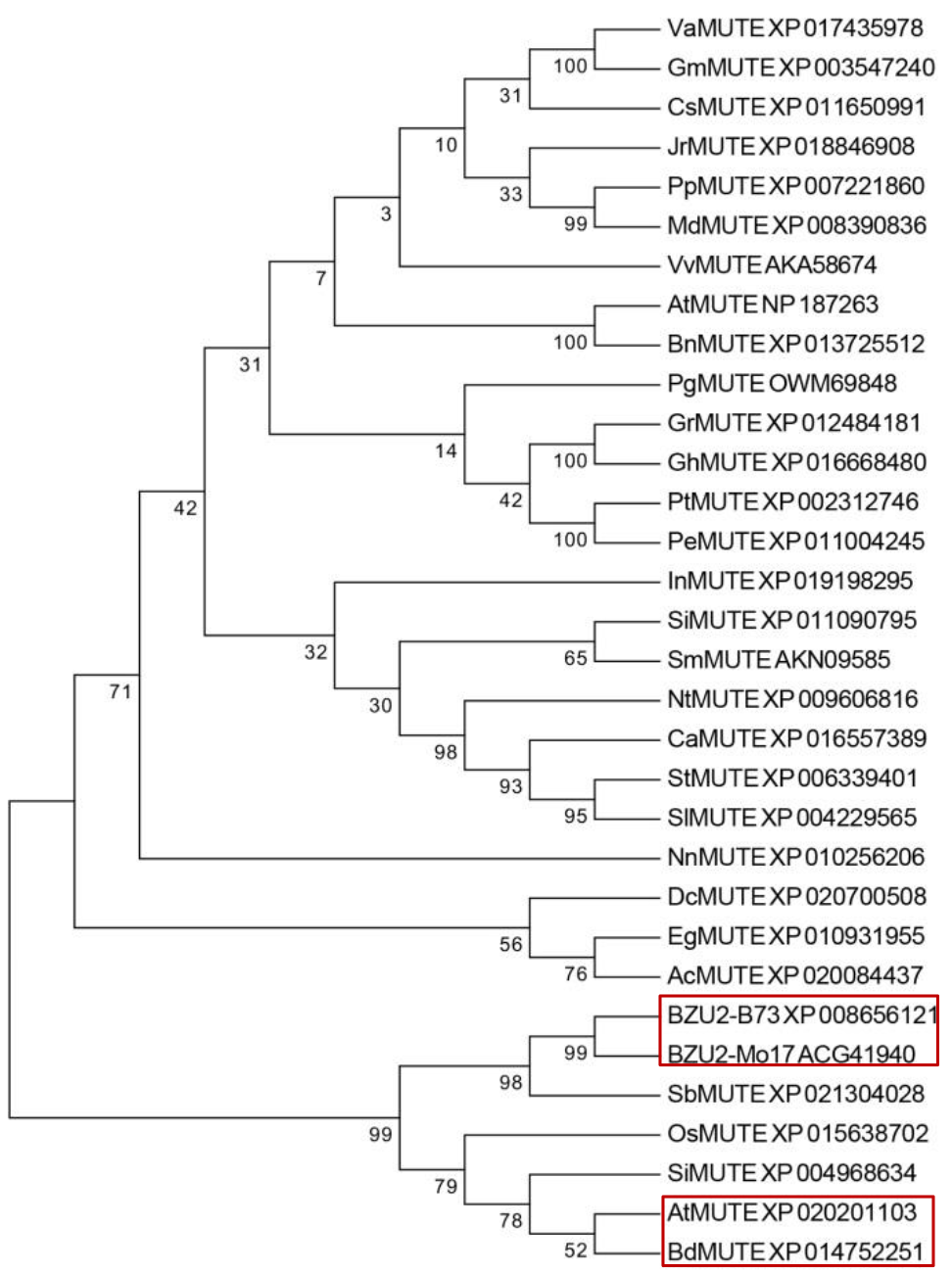

Supplement: S3 Fig — The numbers above or below the branches are the bootstrap values from 5,000 replicates. BZU2/ZmMUTE, AtMUTE and BdMUTE are boxed. NCBI reference sequence numbers are indicated behind the gene names. The species of origin of the MUTE are indicated by the abbreviation preceding the gene names: Ac, Ananas comosus; At, Aegilops tauschii; At, Arabidopsis thaliana; Bd, Brachypodium distachyon; Bn, Brassica napus; Ca, Capsicum annuum; Cs, Cucumis sativus; Dc, Dendrobium catenatum; Eg, Elaeis guineensis; Gh, Gossypium hirsutum; Gm, Glycine max; Gr, Gossypium raimondii; In, Ipomoea nil; Jr, Juglans regia; Md, Malus domestica; Nn, Nelumbo nucifera; Nt, Nicotiana tomentosiformis; Os, Oryza sativa; Pe, Populus euphratica; Pg, Punica granatum; Pp, Prunus persica; Pt, Populus trichocarpa; Sb, Sorghum bicolor; Si, Sesamum indicum; Si, Setaria italica; Sl, Solanum lycopersicum; Sm, Salvia miltiorrhiza; St, Solanum tuberosum; St, Solanum tuberosum; Va, Vigna angularis; Vv, Vitis vinifera; Zm, Zea mays. (TIF) [file pgen.1008377.s003.tif]

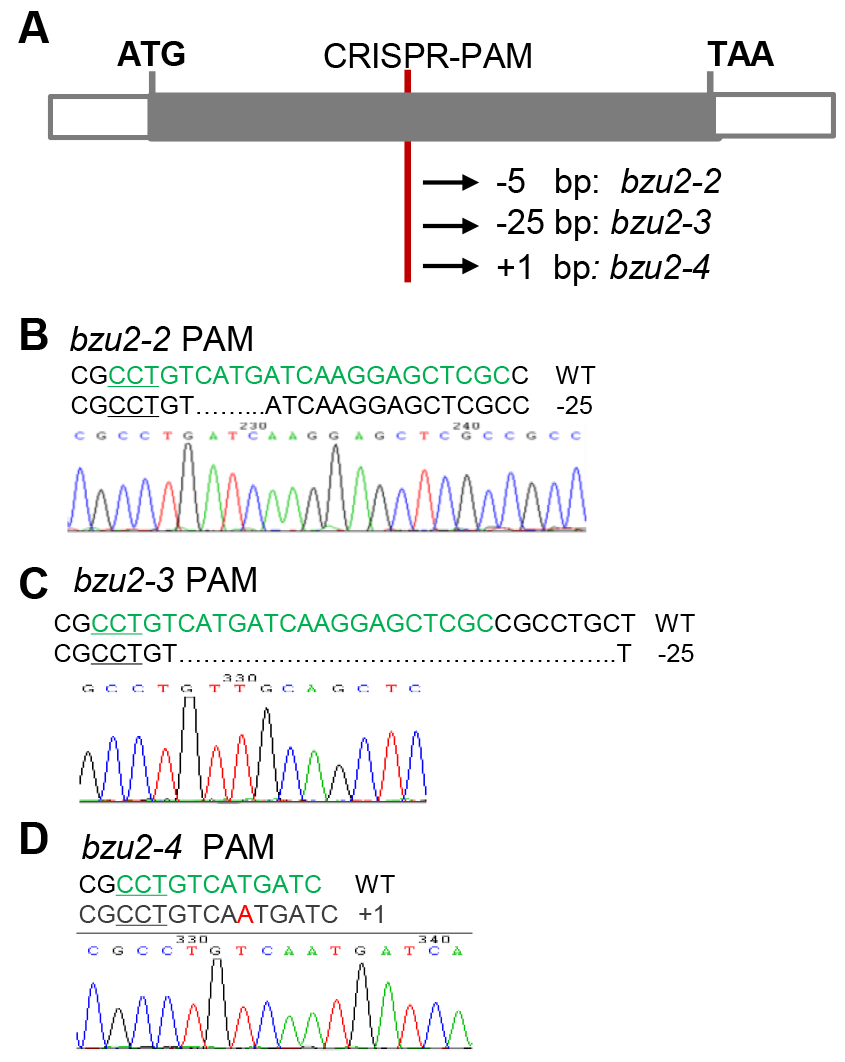

Supplement: S4 Fig — (A) Diagram representing the gRNA target used in the CRISPR/Cas9 system to generate bzu2 mutants. The PAM sequence is located at position +289 nt in BZU2. (B-D) Sequencing confirmation of bzu2 mutants. The mutations in bzu2-2 (B), bzu2-3 (C) and bzu2-4 (D) in the T0 generation. (TIF) [file pgen.1008377.s004.tif]

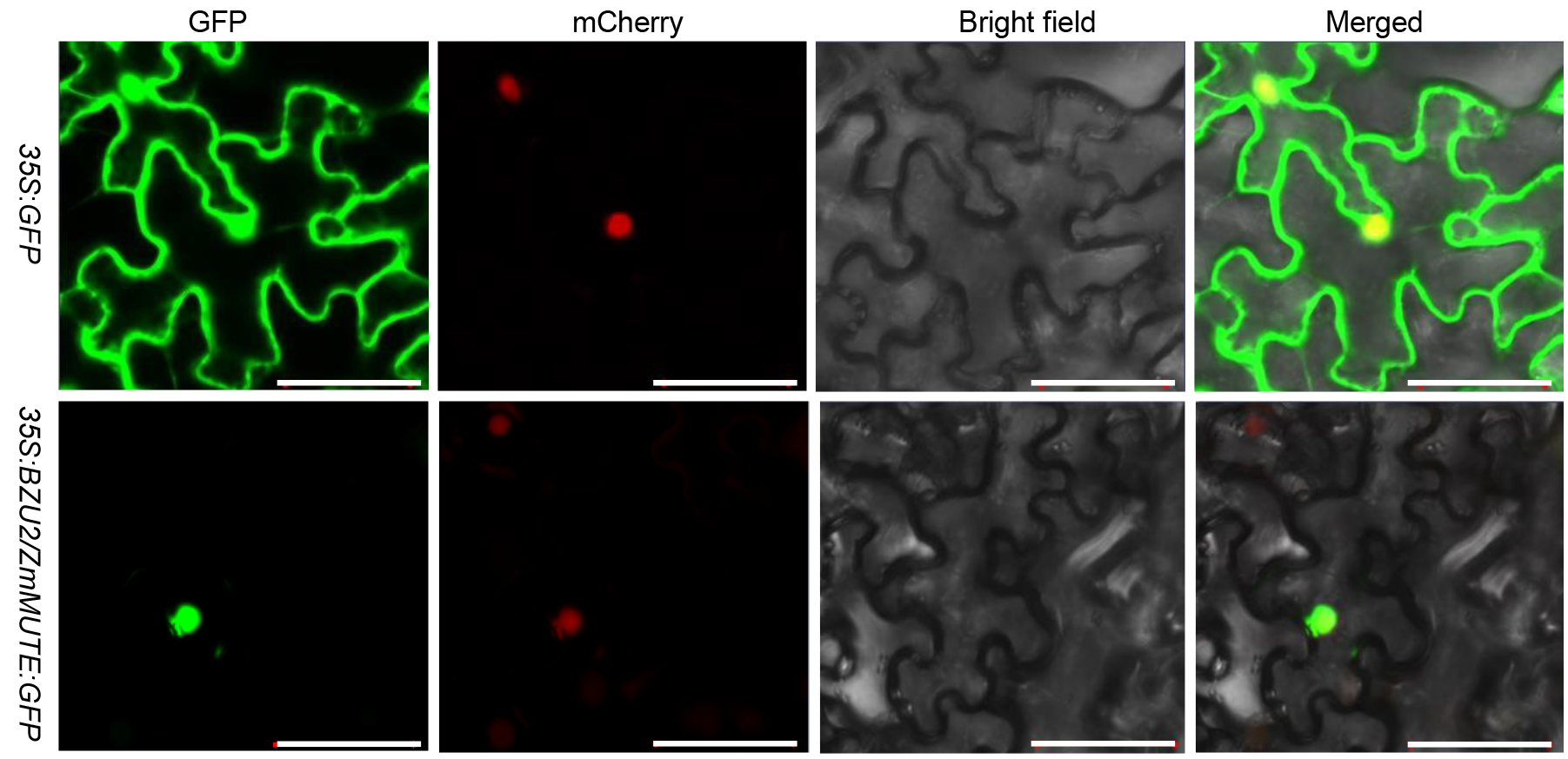

Supplement: S5 Fig — 35S:GFP and 35S:BZU2/ZmMUTE-GFP were transiently expressed in tobacco leaves. Green and red fluorescence was imaged using confocal microscopy 24 h after Agrobacterium-mediated infiltration. 35S:H2B-mCherry serves as a nuclear marker. Scale bars, 50 μm. (TIF) [file pgen.1008377.s005.tif]

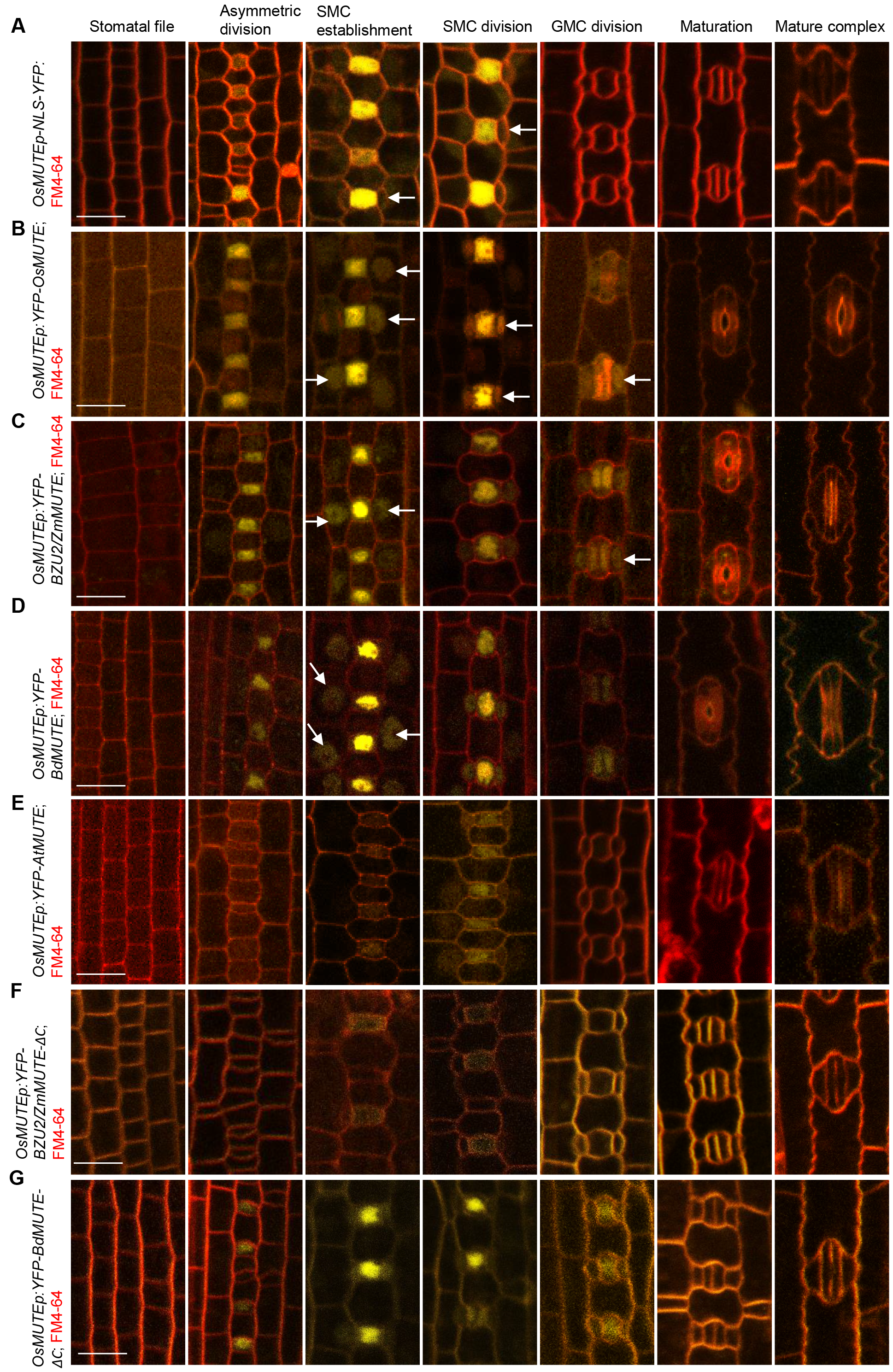

Supplement: S6 Fig — Confocal images of OsMUTEp:NLS-YFP and OsMUTEp:YFP-MUTE expression (yellow) with FM4-64 counterstaining to visualize the outlines of the plasma membranes (red) at the bases of the second and third leaves. White arrows in (A) to (D) indicate the SMC and SC. (A) Expressions of OsMUTEp:NLS-YFP which is only located in the nuclei of GMCs; after the formation of SCs, the signal disappeared from mature guard cells and subsidiary cells. (B) OsMUTEp:YFP-OsMUTE expression at the different stages of stomatal development. (C) OsMUTEp:YFP-ZmMUTE expression at the different stages of stomatal development. OsMUTEp:YFP-ZmMUTE starts to be expressed in the early GMCs, reaching its peak in GMCs, but also being expressed in SMCs. The YFP signal is maintained until after GMC division, finally disappearing during stomatal maturation. (D) OsMUTEp:YFP-BdMUTE expression in different stages of stomatal development. (E) OsMUTEp:YFP-AtMUTE expression at different stages of stomatal development. The fluorescence of YFP-AtMUTE was very weak in the early GMCs, and was not detected in SMCs. Confocal images of OsMUTEp:YFP-ZmMUTE-ΔC (F) and OsMUTEp:YFP- BdMUTE-ΔC (G) show that YFP-ZmMUTE-ΔC and YFP-BdMUTE-ΔC are only found in the early GMCs, and not in the SMCs and young SCs at the base of the second and third leaves. All images are from second or third leaf segments of the T0 (B, C, D and E) and T1 (A, F and G) generations of transgenic rice. Scale bars, 10 μm. (TIF) [file pgen.1008377.s006.tif]

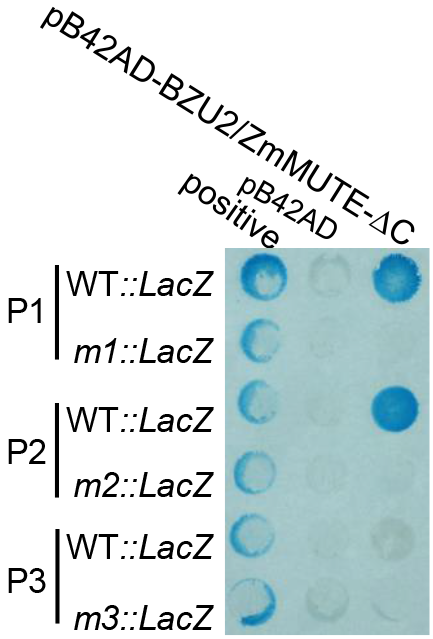

Supplement: S7 Fig — (TIF) [file pgen.1008377.s007.tif]

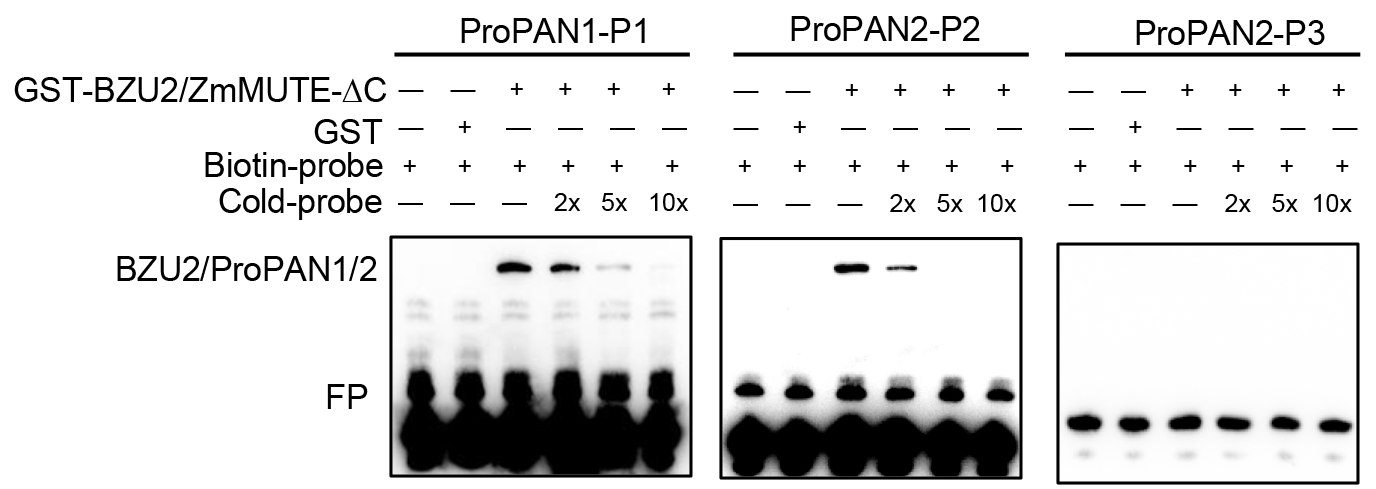

Supplement: S8 Fig — EMSA was performed with probes that were biotin-labeled (biotin probe) or that were unlabeled (cold probe), E-box-containing DNA fragments (top panel) and recombinant BZU2/ZmMUTE-ΔC protein; specific combinations are shown above the autoradiograph. Unlabeled fragments were added gradually in 2-, 5- or 10- fold excess as indicated. (TIF) [file pgen.1008377.s008.tif]

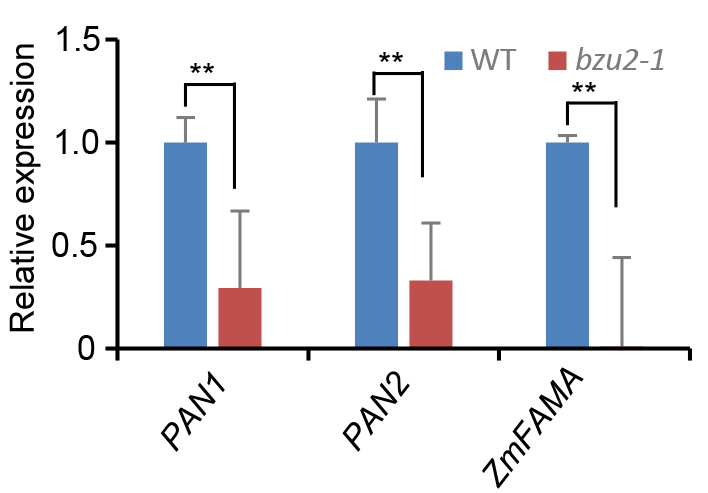

Supplement: S9 Fig — RNA was extracted from leaf segments representing the region up to ~1.5 cm from the leaf bases of the second and third leaves (taken 4 days after germination, and at the point of second leaf emergence). Transcript level is decreased in the bzu2-1 mutant as compared to the wild-type. RT-qPCR values are expressed as the mean ± SD compared to that of the internal control (ZmUbiquitin 2). Error bars indicate SD, n = 3, Student’s t test, **P<0.01. Assays were done with triplicate repeats. (TIF) [file pgen.1008377.s009.tif]

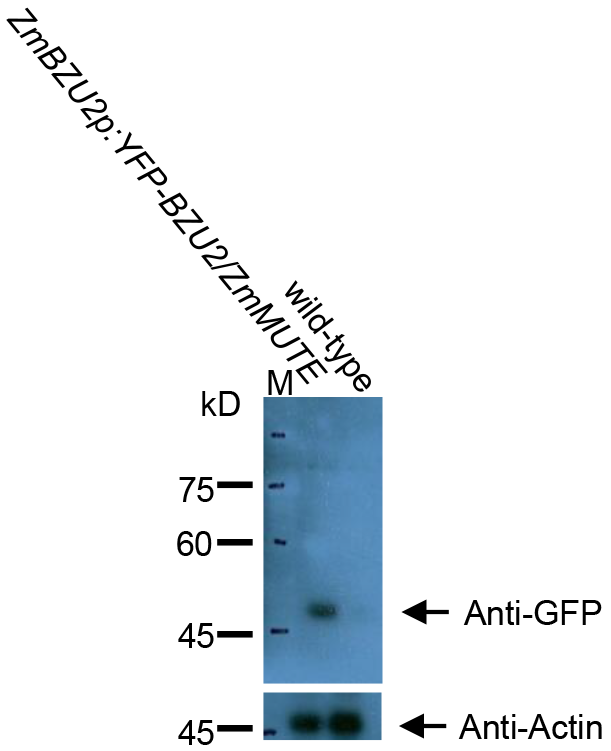

Supplement: S10 Fig — 20 μg of total protein taken from the second and third leaf bases of 8-day-old seedlings of BZU2/ZmMUTEp:YFP-BZU2/ZmMUTE transgenic plants and wild-type, was separated by electrophoresis. The corresponding blot was incubated in primary antibody (anti-GFP, ab290, Abcam) at a dilution 1:10,000, actin being used as a control. (TIF) [file pgen.1008377.s010.tif]

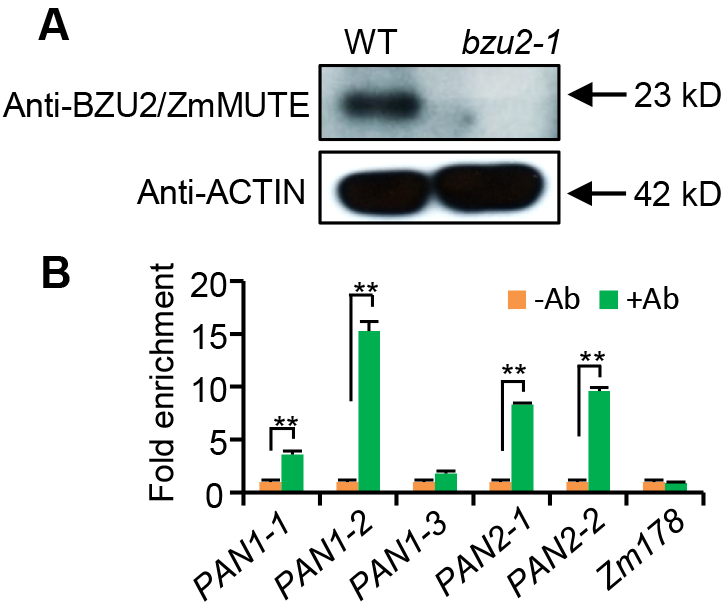

Supplement: S11 Fig — (A) 20 μg of total protein taken from the second and third leaf bases of 8-day-old wild-type and bzu2-1 mutant seedlings. Western blot results indicate that BZU2/ZmMUTE cannot be detected in the bzu2-1 mutant. (B) ChIP-qPCR results showing that the promoter fragments of PAN1 and PAN2 can be amplified from the immunoprecipitation pulled down by the anti-BZU2/ZmMUTE antibody. The sequences used for ChIP-qPCR contain E-box cis-elements from within the PAN1 and PAN2 promoter regions. Samples were harvested for the chromatin immunoprecipitation (ChIP) experiment taken from a region extending ~ 1.5 cm from the base of the second and third leaves of 8-day-old seedlings with (+Ab) or without (-Ab) addition of an anti-BZU2/ZmMUTE antibody. Zm178 (GRMZM2G134178), a gene not involved in stomatal development, was used as a negative control. Error bars indicate SD, n = 3, Student’s t test, **P<0.01. (TIF) [file pgen.1008377.s011.tif]
